# Energetics of sinusoidal exercise below and across critical power and the effects of fatigue

**DOI:** 10.1007/s00421-023-05410-1

**Published:** 2024-01-19

**Authors:** Marta Borrelli, Sheida Shokohyar, Susanna Rampichini, Paolo Bruseghini, Christian Doria, Eloisa Guglielmina Limonta, Guido Ferretti, Fabio Esposito

**Affiliations:** 1 Department of Biomedical Sciences for Health, Università Degli Studi Di Milano, Via Giuseppe Colombo 71, 20133 Milan, Italy; 2 Department of Molecular and Translational Medicine, University of Brescia, Brescia, Italy; 3 IRCCS Ospedale Galeazzi - Sant’Ambrogio, Via Cristina Belgioioso, 173, 20157 Milan, Italy

**Keywords:** Oxygen uptake, Oxygen debt, Blood lactate, Time delay, Sinusoid amplitude, Heart rate

## Abstract

**Purpose:**

Previous studies investigating sinusoidal exercise were not devoted to an analysis of its energetics and of the effects of fatigue. We aimed to determine the contribution of aerobic and anaerobic lactic metabolism to the energy balance and investigate the fatigue effects on the cardiorespiratory and metabolic responses to sinusoidal protocols, across and below critical power (CP).

**Methods:**

Eight males (26.6 ± 6.2 years; 75.6 ± 8.7 kg; maximum oxygen uptake 52.8 ± 7.9 ml·min^−1^·kg^−1^; CP 218 ± 13 W) underwent exhausting sinusoidal cycloergometric exercises, with sinusoid midpoint (MP) at CP (CP_ex_) and 50 W below CP (CP-50_ex_). Sinusoid amplitude (AMP) and period were 50 W and 4 min, respectively. MP, AMP, and time-delay (*t*_*D*_) between mechanical and metabolic signals of expiratory ventilation (${\dot{V}}_{E}$), oxygen uptake (${\dot{V}}_{{{\text{O}}}_{2}}$), and heart rate (${f}_{H}$) were assessed sinusoid-by-sinusoid. Blood lactate ([La^−^]) and rate of perceived exertion (RPE) were determined at each sinusoid.

**Results:**

${\dot{V}}_{{{\text{O}}}_{2}}$ AMP was 304 ± 11 and 488 ± 36 ml·min^−1^ in CP_ex_ and CP-50_ex_, respectively. Asymmetries between rising and declining sinusoid phases occurred in CP_ex_ (36.1 ± 7.7 vs. 41.4 ± 9.7 s for ${\dot{V}}_{{{\text{O}}}_{2}}$ *t*_*D* up_ and *t*_*D* down_, respectively; *P* < 0.01), with unchanged *t*_*D*_*s*. ${\dot{V}}_{{{\text{O}}}_{2}}$ MP and RPE increased progressively during CP_ex_. [La^−^] increased by 2.1 mM in CP_ex_ but remained stable during CP-50_ex_. Anaerobic contribution was larger in CP_ex_ than CP-50_ex_.

**Conclusion:**

The lower aerobic component during CP_ex_ than CP-50_ex_ associated with lactate accumulation explained lower ${\dot{V}}_{{{\text{O}}}_{2}}$ AMP in CP_ex_. The asymmetries in CP_ex_ suggest progressive decline of muscle phosphocreatine concentration, leading to fatigue, as witnessed by RPE.

**Supplementary Information:**

The online version contains supplementary material available at 10.1007/s00421-023-05410-1.

## Introduction

The history of the energetics of muscular exercise concerns mostly the study of the cardiorespiratory and metabolic responses to square-wave, constant-power submaximal work (di Prampero 1981; Whipp and Ward 1982; Poole and Jones 2012; Ferretti 2015; Ferretti et al. 2022). In a square-wave transition from rest to light or moderate exercise, during which no lactate accumulation occurs, the time constant of the primary component of the bi-exponential pulmonary oxygen uptake (${\dot{V}}_{{{\text{O}}}_{2}}$) kinetics (Poole and Jones 2012; Ferretti 2015) corresponds to that of phosphocreatine breakdown (Binzoni et al. 1992; Rossiter et al. 2002). In this type of exercise, the rest-to-exercise transition (on-phase) and exercise-to-rest transition upon exercise cessation (off-phase) show a symmetrical response (Özyener et al. 2001). In contrast, when a square-wave exercise is performed from rest to the heavy exercise domain, respiratory oxygen supply becomes too slow to match the increase in muscle metabolic rate, leading to lactate accumulation during the on-phase (Cerretelli et al. 1979). Due to anaerobic metabolism contribution to energy supply in this transient, asymmetries between the two phases appear, with a longer time constant in the on- than in the off-phase (Paterson and Whipp 1991; Özyener et al. 2001). In this scenario, the appearance of the so-called slow component, accompanied by a continuous increase in blood lactate accumulation, carries along a furtherly accentuated asymmetric pattern between the on- and off-phases of the ${\dot{{\text{V}}}}_{{{\text{O}}}_{2}}$ kinetics (Poole et al. 1994; Jones et al. 2011; Ferretti 2015).

Despite being a convenient experimental model for laboratory studies, square-wave exercise is far away from actual exercise as performed in real life, wherein continuous oscillatory changes in workload occur. To simulate workload oscillations under controlled laboratory conditions, Wigertz (1970) proposed to study exercises in which the mechanical power varied continuously following a sinusoidal function. When we investigate sinusoidal exercise patterns with a sufficiently long sinusoid period (*T*), we should observe a sinusoidal ${\dot{V}}_{{{\text{O}}}_{2}}$ response, with a time lag with respect to the mechanical sinusoidal pattern equal to the time constant of the primary phase of the ${\dot{V}}_{{{\text{O}}}_{2}}$ kinetics (Casaburi et al. 1977; Girardi et al. 2021a; Ferretti 2023). In spite of this, the number of studies so far devoted to an analysis of the cardiorespiratory and metabolic responses to sinusoidal exercise has been relatively small (Wigertz 1970; Casaburi et al. 1977; Bakker et al. 1980; Fukuoka and Ikegami 1990; Haouzi et al. 1993; Fukuoka et al. 1997; Nicolò et al. 2018; Girardi et al. 2021b). In particular, only two studies have investigated the ${\dot{V}}_{{{\text{O}}}_{2}}$ response to sinusoid-wave work rates in two different exercise domains, one in healthy humans (Haouzi et al. 1993), the other in patients affected by chronic obstructive pulmonary disease (Porszasz et al. 2013). Some studies analyzed the effects of varying the T on the patterns followed by cardiorespiratory and metabolic variables during sinusoidal work rate in homogeneously aerobic exercise (light-moderate exercise domain) only, either by simulation (Girardi et al. 2021a) or experimentally (Casaburi et al. 1977; Bakker et al. 1980; Fukuoka and Ikegami 1990).

None of these authors attempted to investigate the energy balance of sinusoidal work rate in different exercise intensity domains. Moreover, all of them studied the cardiorespiratory and metabolic response to sinusoidal exercise work rate of relatively short duration only, without any exploration of the possible effects of peripheral fatigue during prolonged exercises under different intensity domains.

Hence, this study aims to: i) provide a comprehensive comparison of the cardiorespiratory and metabolic responses to two different sinusoidal exercises across and below the critical power (CP), taken as the uppermost limit of fully aerobic exercise (Poole et al. 2016); ii) analyze, from the obtained data, the contribution of aerobic metabolism to the energy balance and quantify blood lactate accumulation, across and below CP; and iii) investigate the effects of fatigue on the cardiorespiratory and metabolic responses to these two sinusoidal protocols up to exhaustion. We hypothesized that, at a given *T*, the sinusoidal exercise across CP may be characterized by a longer time delay between the mechanical and the cardiorespiratory sinusoids (*t*_*D*_*s*) compared to that below CP, especially in the up-phase. This difference in *t*_*D*_*s* may be associated with asymmetries introduced by lactate accumulation during the exercise transient in the former case. This would imply that the contribution of anaerobic metabolism to the energy balance of sinusoidal exercises of equal work rate sinusoid amplitude (AMP) should be larger across than below CP, due to the important role of anaerobic lactic metabolism. Lastly, during exhausting exercise, we expected a drift of the cardiorespiratory and metabolic responses to sinusoidal exercise across, but not below CP.

## Materials and methods

### Participants

Twenty participants were enlisted for inclusion in the experiments. Five of them dropped out for personal reasons, seven were not able to complete the study for different reasons associated with the complexity of the protocol. Therefore, eight physically active male cyclists (age: 26.6 ± 6.2 years; body mass: 75.6 ± 8.7 kg; stature: 1.81 ± 0.04 m; maximum oxygen uptake, ${\dot{V}}_{{{\text{O}}}_{2}{\text{max}}}$: 52.8 ± 7.9 ml∙min^−1^·kg^−1^; mean ± standard deviation) completed this protocol. None of them had any upper or lower limb disease nor cardiorespiratory pathologies. After being fully informed on the purpose of the investigation, the experimental design, and procedures, all the participants provided their written informed consent. The study was approved by the ethics committee of the University of Milan (#77/20) and was performed in accordance with the principles of the latest version of the Declaration of Helsinki.

### Experimental design

Participants reported to the laboratory eight times, with at least 48-h in between. After a first visit for familiarization and anthropometric measurements, on a second occasion participants underwent incremental exercise testing on the cycle ergometer to determine their ${\dot{V}}_{{{\text{O}}}_{2}{\text{max}}}$ and maximum mechanical aerobic power (${\dot{W}}_{{\text{max}}}$) (Adami et al. 2013). During visits 3–6, they performed exhausting square-wave workloads to determine the CP. In visits 7–8, the volunteers performed randomly two sinusoidal tests of different exercise intensity domains until exhaustion, as detailed below.

### Experimental procedures

All the experimental sessions were conducted in a climate-controlled laboratory, with constant temperature of 20 ± 1 °C and relative humidity 50 ± 5%. Tests were performed approximately at the same time of the day to minimize any possible bias induced by circadian rhythms. On the day of the tests, participants were asked to abstain from caffeine and any other similar beverages for at least 12 h and to refrain from intense physical exercise for at least 24 h prior to testing. During the tests, participants were verbally encouraged and strongly motivated by operators to attain their best performance. In all tests, exercise cessation was established when the participant failed to maintain the cadence within the 5 rpm of imposed range for more than 5 s.

#### Familiarization and anthropometric assessment (visit 1)

In the first session, the participants were equipped with a face mask and wearable devices to familiarize with the equipment for cardiopulmonary testing. Moreover, saddle height, handlebar angle of inclination, and foot position over pedal were adjusted individually and maintained during all the subsequent tests. Lastly, participant’s body mass and stature were, respectively, measured to the nearest 0.1 kg and 1 cm, by a mechanical scale equipped with a stadiometer (Asimed, Samadell; Barcelona).

#### Incremental exercise test (visit 2)

On the second occasion, the ${\dot{V}}_{{{\text{O}}}_{2}{\text{max}}}$ and its corresponding ${\dot{W}}_{{\text{max}}}$ were determined through a step-wise incremental test (Adami et al. 2013). After 3 min of baseline recordings and 4 min of warm-up cycling at 100 W, work rate was increased by 25 W every 2 min until exhaustion.

Blood lactate concentration [La^−^] was assessed at baseline, at the end of each work rate, and at minute 1, 3, and 5 of recovery. At the same time of blood sample collection, the rate of perceived exertion (RPE) was asked on a general (RPE_GEN_; Borg 6–20), respiratory, and muscular (RPE_MUSC_ and RPE_RESP_, respectively; CR-10) standpoints. During all tests, participants were asked to keep the cadence between 90 and 100 rpm, because spontaneously adopted in cyclists to minimize local muscle stress and improve effectiveness of the skeletal-muscle pump in facilitating venous return to the heart (Lucia et al. 2004).

#### Critical power assessment (visits 3–6)

On different days, to determine CP, four exhausting square-wave tasks at different intensities (90–115% ${\dot{W}}_{{\text{max}}}$) were administered (Hill 1993; Mattioni Maturana et al. 2018), so that the time-to-exhaustion was in the range between 2 and 20 min (Morton 2006; Vinetti et al. 2019).

#### Sinusoidal work rate tests (visits 7–8)

After CP assessment, volunteers randomly performed two exhausting sinusoidal tasks at different work rates. The protocols involved 3 min of baseline recordings, followed by a warm-up of 3 min at 50 W and 3 min at CP, after which the sinusoidal exercise began in its downward midpoint crossing toward the nadir. The warm-up at CP was chosen to standardize the starting point of both protocols.

The mechanical power, as a function of time (*t*), fluctuated in a sinusoidal fashion according to:

$$f\left(t\right)={\text{MP}}+{\text{AMP}}\cdot {\text{sin}}\left(\frac{2\pi }{T}t\right)$$

where, *T* is the period of the sinusoid, MP, the sinusoid midpoint, is the value around which the sinusoidal function oscillates, and AMP is the amplitude between MP and either the nadir or the zenith of the sinusoid.

**Fig. 1:**
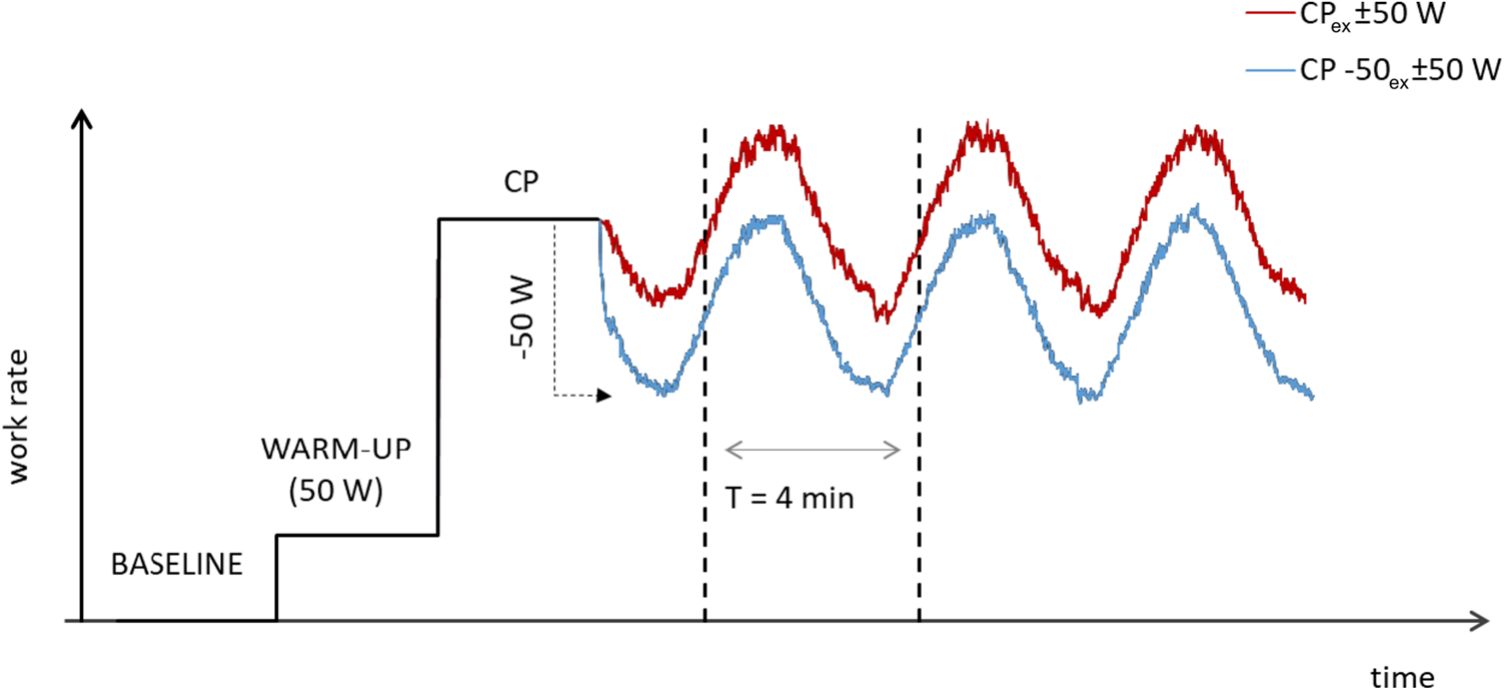
Illustration of the two original sinusoidal work rates: CP_ex_ (*red line*) and CP-50_ex_ (*blue line*). *CP* critical power, *MP* midpoint, *T* period (color figure online)

Based on previous study trials, the sinusoidal protocols had the MP for power set at or 50 W below the CP (CP_ex_ and CP-50_ex_, respectively). In both protocols, AMP and *T* were 50 W and 4 min, respectively (Fig. 1), so that the overall power change between nadir and zenith was 100 W. The investigated AMP was selected as absolute power, to make the energy balance assessment possible. The 4-min *T* was chosen because it is long enough to accommodate the primary component and at the same time long enough to reveal any impacts of a slower component (Haouzi et al. 1993).

[La^−^] and RPE values (RPE_GEN_, RPE_MUSC_ and RPE_RESP_, respectively) were determined at baseline, at the end of CP warm-up, at the end of each cycle (corresponding to downward MP crossing), and at minute 1, 3, and 5 of recovery.

### Measurements

Tests were performed on an electro-mechanically braked cycle ergometer (mod. 839E, Monark, Sweden). During the experiments, the work rate and cadence were continuously recorded. Expiratory ventilation (${\dot{V}}_{E}$), ${\dot{V}}_{{{\text{O}}}_{2}}$, pulmonary carbon dioxide output (${\dot{V}}_{{{\text{CO}}}_{2}}$), respiratory rate (${f}_{R}$), tidal volume (${V}_{T}$), alveolar carbon dioxide pressure (${P}_{{{\text{ACO}}}_{2}}$), and respiratory exchange ratio were measured on a breath-by-breath basis by a metabolic unit consisting of a turbine flowmeter, a zirconium oxygen sensor, and an infrared CO_2_ meter (Quark b^2^, Cosmed, Rome, Italy). According to manufacturer’s instructions, the turbine and gas analyzers were calibrated before each test by means of a 3-l syringe (mod. 5530, Hans-Rudolph, Shawnee, KS, USA) and a certified gas mixture of known concentration (16% O_2_, 5% CO_2_, balance N_2_), respectively. ${\dot{V}}_{{{\text{O}}}_{2}}$ and ${\dot{V}}_{{{\text{CO}}}_{2}}$ were computed using the Auchincloss algorithm (Auchincloss et al. 1966). Heart rate (${f}_{H}$) was continuously acquired (S810, Polar Electro Oy, Kempele, Finland). Lastly, 20 μl arterialized blood samples were taken from the ear lobe and analyzed by an enzymatic-amperometric system (Labtrend, Bio Sensor Technology GmbH, Berlin, Germany) to determine [La^−^].

### Data analysis

All the experimental data were analyzed offline. The respiratory and gas exchange responses were edited of spurious breaths that resulted from swallowing, coughing, sighing or premature ending of breath, by deleting values outside three standard deviation (SD) from the local mean (Lamarra et al. 1987).

From the incremental step-wise test, ${\dot{V}}_{{{\text{O}}}_{2}{\text{max}}}$ was identified from the plateau in the relationship between ${\dot{V}}_{{{\text{O}}}_{2}}$, selected by averaging the last 30 s of each workload, and mechanical power ($\dot{W}$). Two subjects did not exhibit a ${\dot{{\text{V}}}}_{{{\text{O}}}_{2}}$ plateau, therefore, the highest value of ${\dot{V}}_{{{\text{O}}}_{2}}$ was considered as ${\dot{V}}_{{{\text{O}}}_{2}{\text{max}}}$. The ${\dot{W}}_{{\text{max}}}$ was determined as the mechanical load corresponding to the intersection between the ${\dot{V}}_{{{\text{O}}}_{2}}$ plateau and the linear relationship between ${\dot{V}}_{{{\text{O}}}_{2}}$ and $\dot{W}$. The lactate accumulation point was estimated by *D*_max_-modified method (Bishop et al. 1998) as being correlated with maximal lactate steady state (MLSS) indicator (Van Schuylenbergh et al. 2004).

To provide an accurate measure of CP, tests shorter than 2 min were not considered in the analysis (Mattioni Maturana et al. 2018). The hyperbolic power–duration relationship was transformed into a linear formulation (Hill 1993), where the y-intercept of the regression line of $\dot{W}$ vs. the inverse of exhaustion time was retained as CP.

The first 2 min of sinusoidal exercise were excluded from analysis to avoid any distortion in the physiological response induced by the transition of the workload intensity from CP (during the 3 min warm-up phase) to MP (sinusoidal phase). The cardiorespiratory and metabolic response was fitted by the sinewave function that minimized the least squared errors through a custom-built software (Matlab 2019b, MathWorks Inc., Natick, USA). AMP, MP, *t*_*D*_*s* of all measured variables were calculated for each sinusoid. The average values of these three parameters from all the completed cycles were also determined. Moreover, the net amount of oxygen taken up within a cycle, reflecting the amount of energy derived from aerobic sources only during a sinusoid (${E}_{{\text{s}},{{\text{O}}}_{2}}$), was computed as the time integral of the ${\dot{V}}_{{{\text{O}}}_{2}}$ variation during the cycle itself. This latter was obtained by subtracting the ${\dot{V}}_{{{\text{O}}}_{2}}$ at the nadir of the cycle from the overall ${\dot{V}}_{{{\text{O}}}_{2}}$ of each breath.

**Fig. 2:**
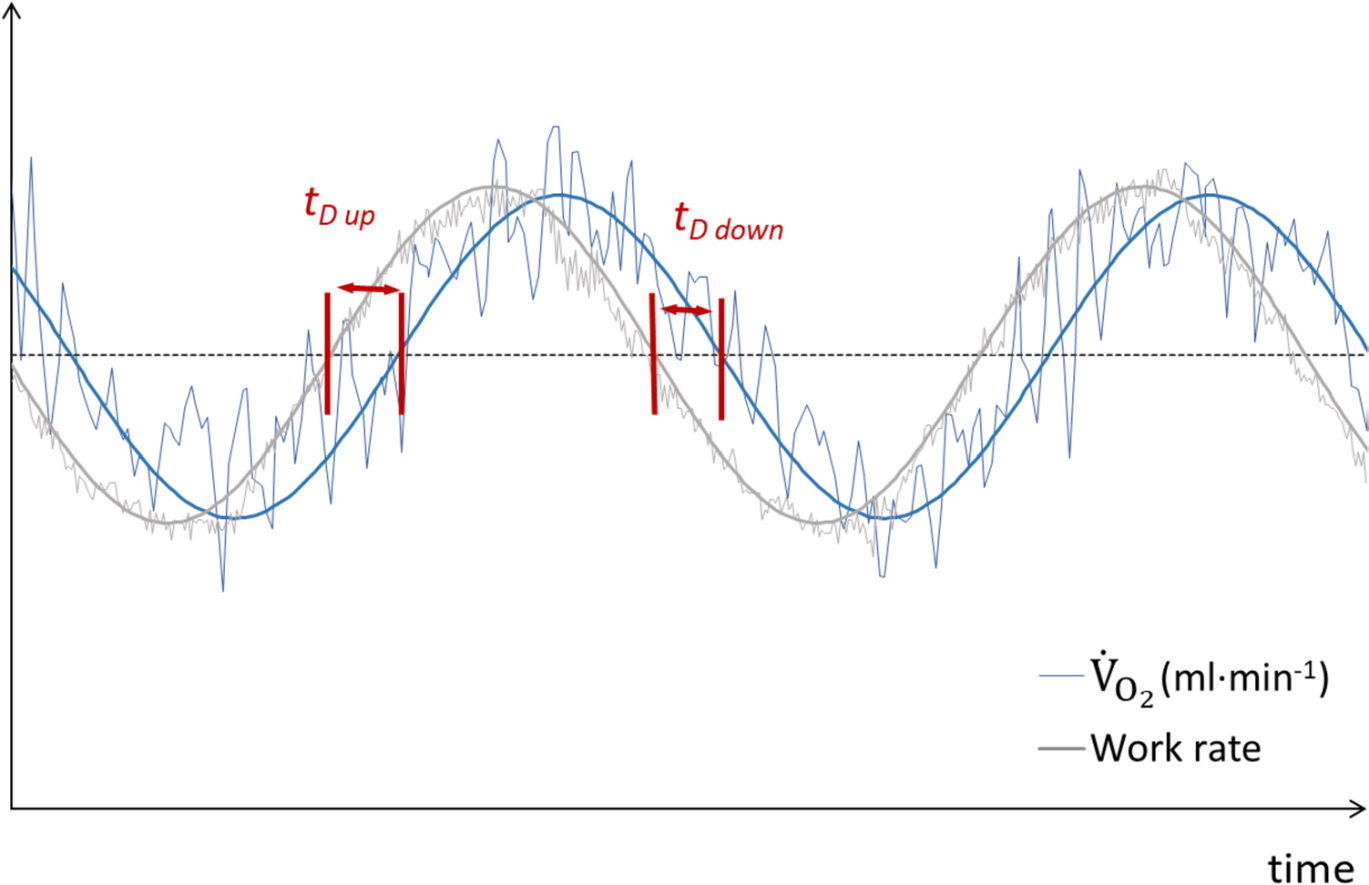
Raw signals of a representative subject during two cycles of a sinusoidal protocol. *Grey line*: work rate; *blue line*: pulmonary oxygen uptake, ${\dot{V}}_{{{\text{O}}}_{2}}$.*t*_*D* up_, delay at upward midpoint (MP) crossing; *t*_*D* down_, delay at downward MP crossing (color figure online)

*t*_*D*_ was determined as the time shift between the mechanical load and the physiological response. Two different *t*_*D*_*s* were determined according to the following hallmarks: i) *t*_*D* up_: upward MP crossing and ii) *t*_*D* down_: downward MP crossing (Fukuoka et al. 1997) (Fig. 2). All these parameters were determined on a cycle-by-cycle basis for all the cardiorespiratory and metabolic variables. In addition, the average value of the investigated variables was also computed over all cycles.

### Statistical analysis

Descriptive statistics were used to characterize the study sample. The Shapiro–Wilk test was applied to check the normal distribution. A two-way analysis of variance for repeated measures (two-way ANOVA RM) assessed the presence of differences in AMP, MP, and *t*_*D*_*s* between the two work rates and among sinusoidal cycles. In the statistical analysis, the minimum number of cycles completed by all subjects, minus one, and the last completed cycle were included. For all pair-wise multiple comparisons, the Bonferroni’s correction test was applied. A paired Student’s *t* test determined the differences between average and all-cycle values and between the two sinusoidal protocols of cardiorespiratory and metabolic variables. Paired Student’s *t* test also established the differences between the number of cycles completed during the two sinusoidal exercises and the difference between *t*_*D* up_ and *t*_*D* down_. The Hedge’s g effect size with CI_95%_ was also calculated and interpreted as follows: 0.00–0.19: trivial; 0.20–0.59: small; 0.60–1.19: moderate; 1.20–1.99: large; ≥ 2.00: very large (Hopkins et al. 2009). All statistical analyses were performed using a statistical software (IBM SPSS Statistics v. 26, Armonk, NY, USA). The significance level was set at *α* < 0.05. Unless, otherwise stated, results are presented as mean ± standard error (SE).

## Results

The main results obtained during the step-wise incremental exercise are shown in Table 1. The mean $\dot{{\text{W}}}$ at the lactate accumulation point and at CP were 205 ± 13 and 218 ± 13 W, respectively. The latter was considered to set individually the sinusoidal exercises intensity.

**Table 1:** Main outcomes of step-wise incremental test at peak of exercise

$\dot{W}$ (W)	266 ± 16
${\dot{V}}_{{{\text{O}}}_{2}}$ (ml·min^−1^)	3971 ± 207
${\dot{{\text{V}}}}_{{{\text{CO}}}_{2}}$ (ml·min^−1^)	4391 ± 202
RER	1.11 ± 0.01
$\it {{\text{f}}}_{{\text{H}}}$ (beats·min^−1^)	186 ± 2
${\dot{V}}_{{\text{E}}}$ (l·min^−1^)	176 ± 5
${f}_{R}$ (breaths·min^−1^)	48.8 ± 2.6
${V}_{T}$ (l)	3.19 ± 0.17
[La^−^] (mM)	9.3 ± 0.8
RPE_GEN_ (u.a.)	18.0 ± 0.5
RPE_MUSC_ (a.u.)	8.6 ± 0.6
RPE_RESP_ (a.u.)	7.4 ± 0.5

Maximal mechanical aerobic power ($\dot{W}$), pulmonary oxygen uptake (${\dot{V}}_{{{\text{O}}}_{2}}$), carbon dioxide production (${\dot{V}}_{{{\text{CO}}}_{2}}$), respiratory exchange ratio (RER), heart rate (${f}_{H}$), expiratory ventilation (${\dot{{\text{V}}}}_{E}$), respiratory rate (${f}_{R}$), tidal volume (${{\text{V}}}_{T}$), blood lactate concentration ([La^−^]), rates of perceived exertion on a general (RPE_GEN_; Borg 6–20), muscular and respiratory (RPE_MUSC_ and RPE_RESP_; CR-10). Data are shown as mean ± standard error (SE)

### Sinusoidal workloads

**Fig. 3:**
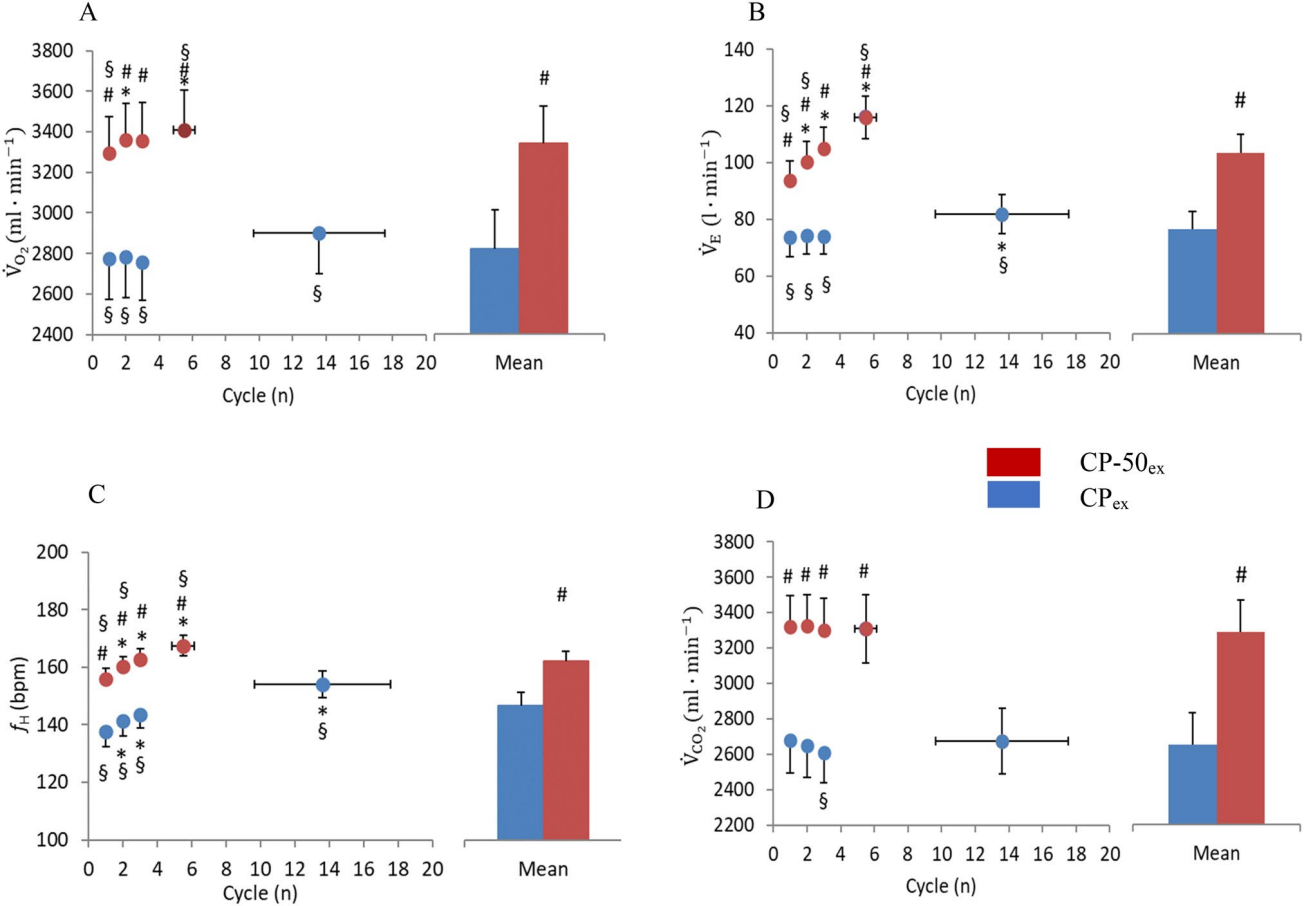
Midpoint (MP) response of pulmonary oxygen uptake, ${\dot{V}}_{{{\text{O}}}_{2}}$ (panel A), ventilation, ${\dot{V}}_{E}$ (panel B), heart rate, ${f}_{H}$ (panel C), carbon dioxide production, ${\dot{V}}_{{{\text{CO}}}_{2}}$ (panel D) to each sinewave (Cycle) of CP_ex_ (*red circles*) and CP-50_ex_ (*blue circles*). The column charts represent the average of all cycles during CP_ex_ (*red bars*) and CP-50_ex_ (*blue bars*). * *P* < 0.05 vs*.* Cycle 1; ^#^*P* < 0.05 vs*.* CP_ex_; ^§^*P* < 0.05 vs. averaged cycles. Data are shown as mean ± standard error (SE) (color figure online)

**Fig. 4:**
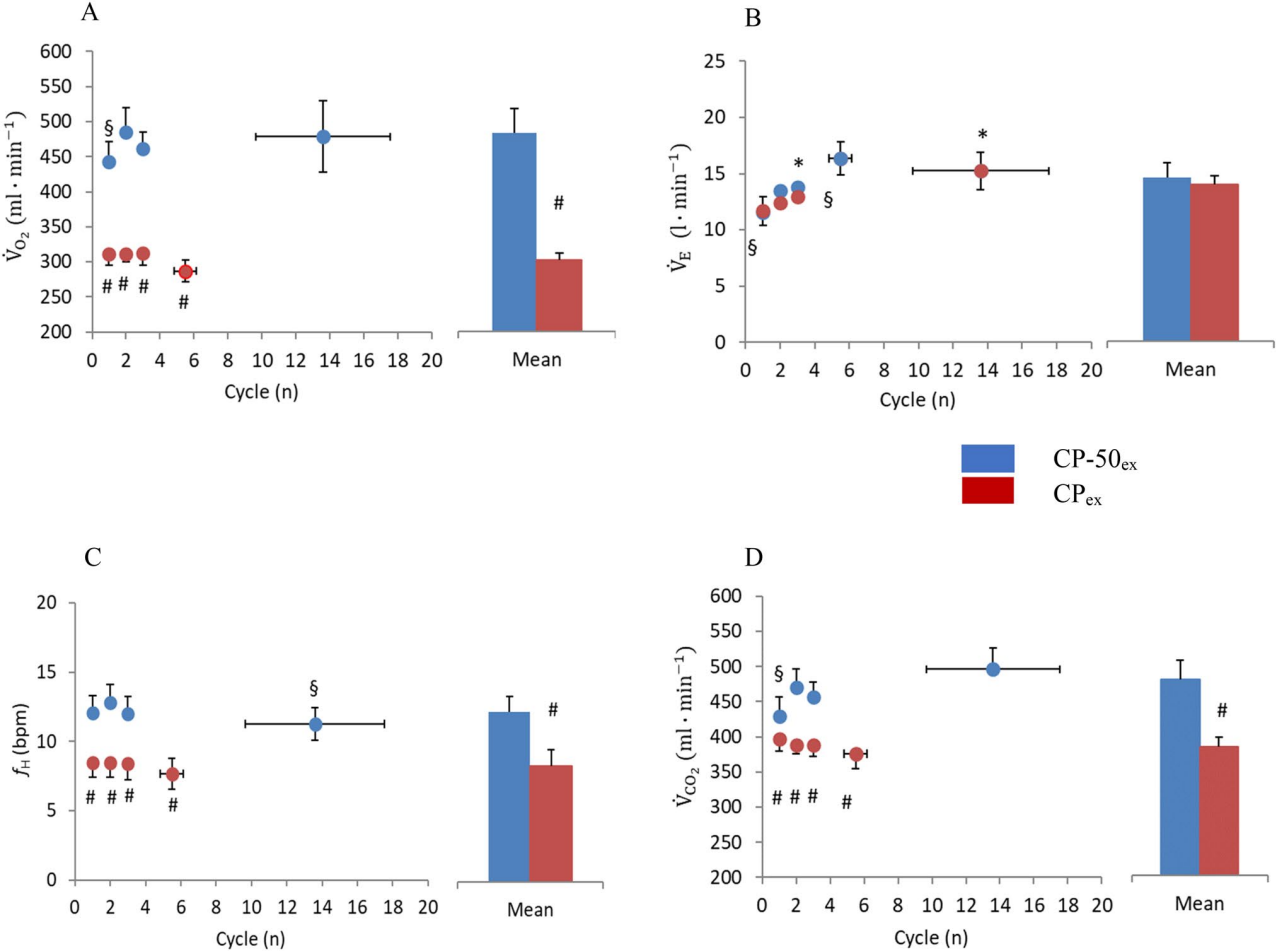
Amplitude (AMP) response between midpoint and zenith of pulmonary oxygen uptake, ${\dot{V}}_{{{\text{O}}}_{2}}$ (panel a), ventilation, ${\dot{V}}_{E}$ (panel b), heart rate, ${f}_{H}$ (panel c), carbon dioxide production, ${\dot{V}}_{{{\text{CO}}}_{2}}$ (panel d) to each sinewave (Cycle) of CP_ex_ (*red circles*) and CP-50_ex_ (*blue circles*). The column charts represent the average of all cycles during CP_ex_ (*red bars*) and CP-50_ex_ (*blue bars*). *P < 0.05 vs. Cycle 1; ^#^*P* < 0.05 vs. CP_ex_; ^§^*P* < 0.05 vs. averaged cycles. Data are shown as mean ± standard error (SE) (color figure online)

**Fig. 5:**
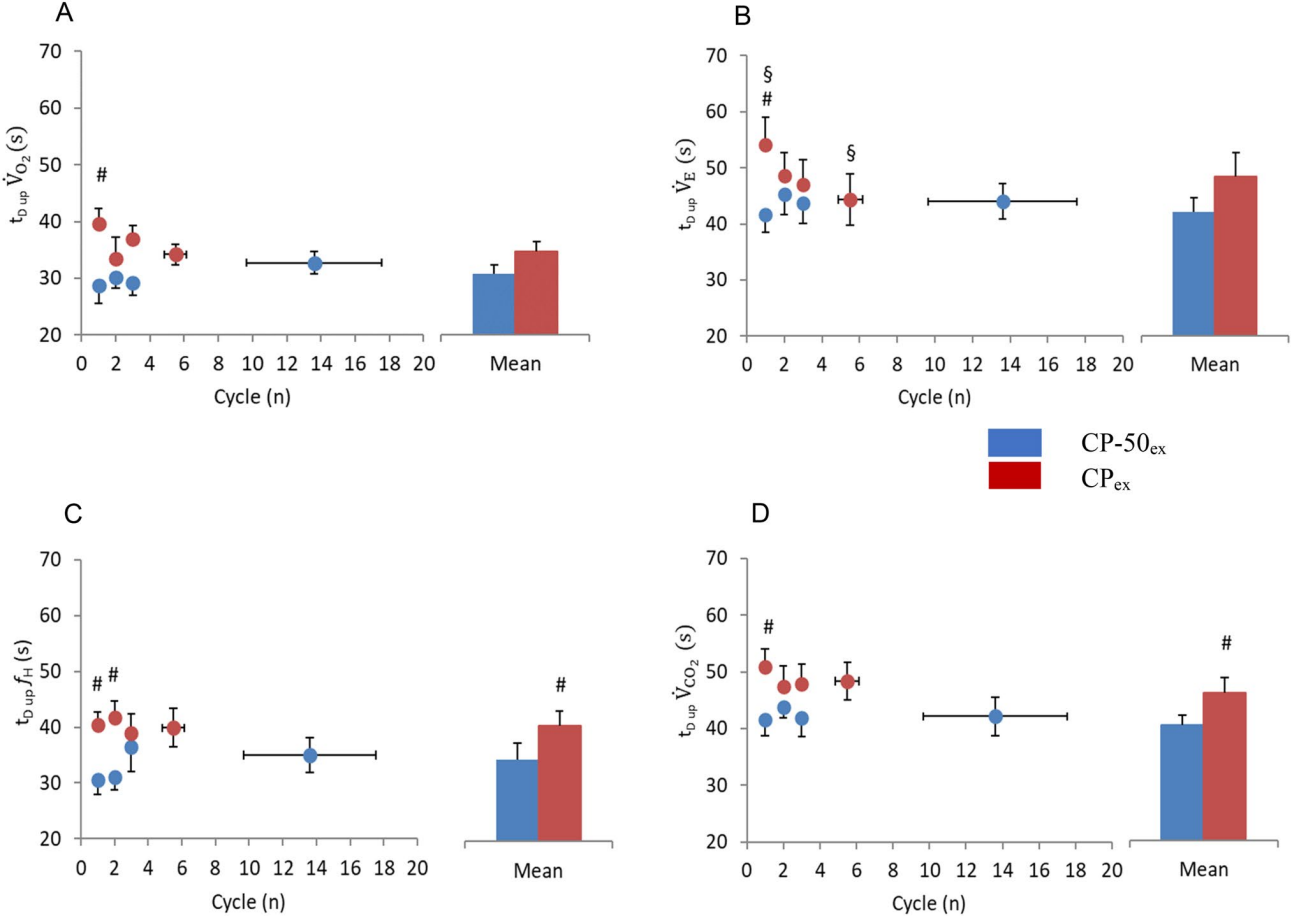
Time delay upward MP crossing (*t*_*D* up_) of pulmonary oxygen uptake, ${\dot{V}}_{{{\text{O}}}_{2}}$ (panel A), ventilation, ${\dot{V}}_{E}$ (panel B), heart rate, ${f}_{H}$ (panel C), carbon dioxide production, ${\dot{V}}_{{{\text{CO}}}_{2}}$ (panel D) to each sinewave (Cycle) of CP_ex_ (*red circles*) and CP-50_ex_ (*blue circles*). The column charts represent the average of all cycles during CP_ex_ (*red bars*) and CP-50_ex_ (*blue bars*). *P < 0.05 vs. Cycle 1; ^#^*P* < 0.05 vs. CP_ex_; ^§^*P* < 0.05 vs. averaged cycles. Data are shown as mean ± standard error (SE) (color figure online)

**Fig. 6:**
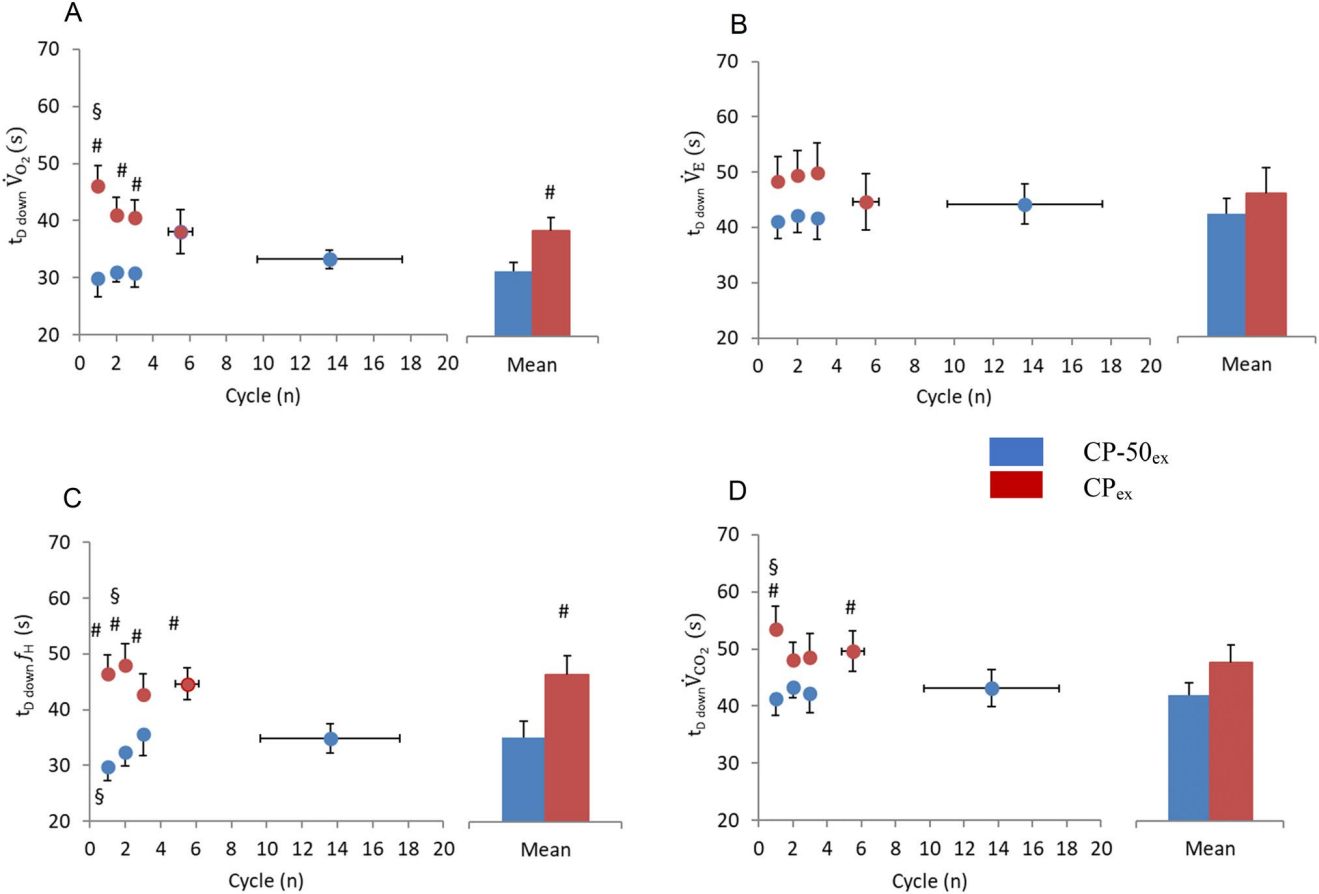
Time delay downward MP crossing (*t*_*D* down_) of pulmonary oxygen uptake, ${\dot{V}}_{{{\text{O}}}_{2}}$ (panel A), ventilation, ${\dot{V}}_{E}$ (panel B), heart rate, ${f}_{H}$ (panel C), carbon dioxide production, ${\dot{V}}_{{{\text{CO}}}_{2}}$ (panel D) to each sinewave (Cycle) of CP_ex_ (*red circles*) and CP-50_ex_ (*blue circles*). The column charts represent the average of all cycles during CP_ex_ (*red bars*) and CP-50_ex_ (*blue bars*). *P < 0.05 vs. Cycle 1; ^#^*P* < 0.05 vs. CP_ex_; ^§^*P* < 0.05 vs. averaged cycles. Data are shown as mean ± standard error (SE) (color figure online)

**Fig. 7:**
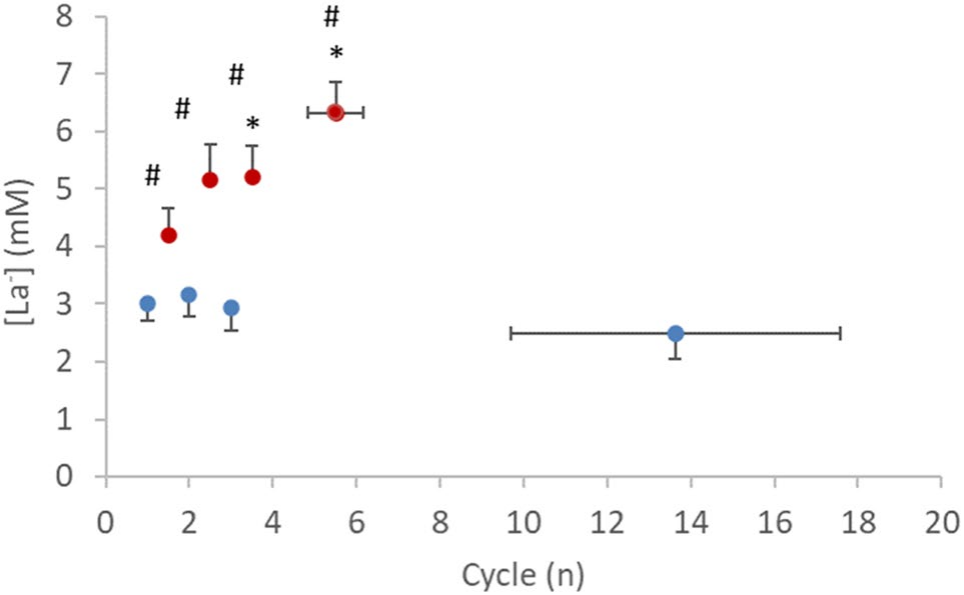
Blood lactate concentration ([La^−^]) to each cycle of CP_ex_ (*red circles*) and CP-50_ex_ (*blue circles*). *P < 0.05 vs. 1; ^#^*P* < 0.05 vs. CP_ex_. Data are shown as mean ± standard error (SE) (color figure online)

The main outcomes related to sinusoidal parameters are shown in Figs. 3, 4, 5, 6, 7. As expected, the number of cycles completed during CP-50_ex_ was higher than that completed during CP_ex_ (13.6 ± 3.9 and 5.5 ± 0.7, respectively; *P* = 0.029; *g* = 0.96, CI_95%_: −0.34 to −76.69). The details about the statistical analysis (the Hedge’s g effect size and CI_95%_) are provided as Supplemental Material. The figures display group-mean responses for the sake of readability. Supplementary Materials include figures depicting also individual data obtained during the experiments.

### Midpoint, MP

In CP-50_ex_, MP of all cardiorespiratory and metabolic variables was lower compared to CP_ex_ in all the cycles and as average (*P* < 0.001). In CP_ex_, ${\dot{V}}_{{{\text{O}}}_{2}}$ MP of the second and the last cycles was higher compared to the first one (*P* = 0.038; *P* = 0.050, respectively), while in CP-50_ex_, ${\dot{V}}_{{{\text{O}}}_{2}}$ MP was invariant across the cycles. At both work rates, the ${\dot{V}}_{{{\text{O}}}_{2}}$ MP of the first and the last sinusoid was different from the average of all cycles.

In CP_ex_, an increment in ${\dot{V}}_{{\text{E}}}$ MP was observed already from the second cycle (*P* < 0.001), while in CP-50_ex_, only the ${\dot{V}}_{{\text{E}}}$ MP of the last cycle was higher than that of the first cycle (*P* = 0.042). In both protocols, the ${\dot{V}}_{{\text{E}}}$ MP of most sinusoids turned out different from the overall average (*P* < 0.05). In both work rates, ${\dot{V}}_{{{\text{CO}}}_{2}}$ MP remained unchanged. For ${f}_{H}$, in contrast, MP progressively increased in both work rates, to become higher than in the first sinusoid from the third sinusoid on (*P* < 0.001). In both protocols, the ${f}_{H}$ MP of most sinusoids turned out different from the overall average (*P* < 0.05).

### Amplitude, AMP

During CP_ex_, lower values of ${\dot{V}}_{{{\text{O}}}_{2}}$ AMP were observed than during CP-50_ex_ for both the single sinusoids (*P* < 0.01) and the average value (*P* = 0.004). In both the work rates, the ${\dot{V}}_{{{\text{O}}}_{2}}$ AMP remained stable for the entire duration of the protocols.

Conversely, no differences in ${\dot{V}}_{E}$ AMP were observed between CP_ex_ and CP-50_ex_ either in single cycles (*P* = 0.92) or in the average (*P* = 0.74). Coherently, within each protocol, we observed a progressive increase in ${\dot{V}}_{E}$ AMP from the first to the last sinusoid (from 11.6 + 1.1 to 15.3 + 1.7 l·min^−1^ in CP-50_ex_, and from 11.7 + 1.3 to 16.4 + 1.5 l·min^−1^ in CP_ex_), despite contradictory statistic outcomes (*P* = 0.040 in the former case and *P* = 0.46 in the latter case). Yet, in CP_ex_, differences were found between the last cycles with respect to the average value (*P* = 0.050).

${\dot{V}}_{{{\text{CO}}}_{2}}$ AMP reported lower values in CP_ex_ than in CP-50_ex_ for the second, third, and last cycles (*P* < 0.05), as well as for the average value (*P* = 0.037). In both work rates, ${\dot{V}}_{{{\text{CO}}}_{2}}$ AMP remained stable for the entire duration of the protocol. Differences with average value were found in the first and the third sinusoids of CP-50_ex_ (*P* = 0.006 and *P* = 0.050, respectively).

In comparison with CP-50_ex_, CP_ex_ was characterized by a lower ${f}_{H}$ AMP for all cycles (*P* < 0.01) and for the average (*P* = 0.004). In CP_ex_ and in CP-50_ex_ ${f}_{H}$ AMP did not change for the entire duration of the protocol.

### Aerobic energy sources, ${E}_{s,{O}_{2}}$

The ${E}_{s,{{\text{O}}}_{2}}$ resulted equal to 2000 ± 125 and 1658 ± 126 ml, in CP-50_ex_ and CP_ex_, respectively. Considering that the duration of a cycle was 4 min, this implies that the corresponding mean ${\dot{V}}_{{{\text{O}}}_{2}}$ above the nadir during a cycle was 500 ± 32 and 415 ± 31 ml∙min^−1^. Since the ${\dot{V}}_{{{\text{O}}}_{2}}$ at the nadir was 2261 ± 200 and 2895 ± 185 ml∙min^−1^ in CP-50_ex_ and CP_ex_, respectively, the mean overall ${\dot{V}}_{{{\text{O}}}_{2}}$ calculated this way (2761 ± 202 and 3310 ± 187 ml∙min^−1^, respectively) did not differ significantly from the determined ${\dot{V}}_{{{\text{O}}}_{2}}$ MP (*P* = 0.64 and 0.83 for CP-50_ex_ and CP_ex_, respectively).

### Time delays, t_*D*_*s*

In CP_ex_, ${\dot{V}}_{{{\text{O}}}_{2}}$ *t*_*D* up_ was longer than in CP-50_ex_ in the first cycle only (*P* = 0.016). A similar tendency appeared for ${\dot{V}}_{E}$ *t*_*D* up_ (*P* = 0.059). As a consequence, no differences between single cycles and average of all cycles appeared for both ${\dot{V}}_{{{\text{O}}}_{2}}$ and ${\dot{V}}_{E}$, except for the last cycles of ${\dot{V}}_{E}$ (*P* = 0.042). ${\dot{V}}_{{{\text{CO}}}_{2}}$ *t*_*D* up_ in CP_ex_ was longer than in CP-50_ex_ in the first cycle only (*P* = 0.021). Similarly, in CP_ex_, ${f}_{H}$ *t*_*D* up_ was longer than during CP-50_ex_ in the first and in the second sinusoids (*P* = 0.002 and *P* = 0.001, respectively) and in the average (*P* = 0.018).

CP_ex_ was characterized by longer ${\dot{V}}_{{{\text{O}}}_{2}}$ *t*_*D* down_ compared to CP-50_ex_ in the first three cycles (*P* < 0.01) and the average (*P* = 0.009). Moreover, in CP_ex_, ${\dot{V}}_{{{\text{O}}}_{2}}$ *t*_*D* down_ of the first cycle was longer than the average (*P* = 0.006). No differences between protocols and with the average values were observed in ${\dot{V}}_{E}$ *t*_*D* down_. In CP_ex_ ${\dot{V}}_{{{\text{CO}}}_{2}}$ *t*_*D* down_ was longer than in CP-50_ex_ in the first and last cycles (*P* = 0.005 and *P* = 0.034, respectively). Moreover, in CP_ex_, a higher value of ${\dot{V}}_{{{\text{CO}}}_{2}}$ *t*_*D* down_ was found in the first cycle compared to the average (*P* = 0.020). Similar patterns were followed by ${f}_{H}$ *t*_*D* down_, except for the fact that the differences between the two protocols appeared for all the cycles (*P* < 0.05), and thus for the average (*P* = 0.0001).

In all cardiorespiratory and metabolic *t*_*D* up_ and *t*_*D* down_, no changes were revealed by the within protocol analysis; indeed, in both sinusoidal exercises, they remained unchanged.

Overall, ${\dot{V}}_{{{\text{O}}}_{2}}$ *t*_*D* up_ was 30.2 ± 6.6 s and ${\dot{V}}_{{{\text{O}}}_{2}}$ *t*_*D* down_ was 31.2 ± 6.3 s in CP-50_ex_. This 1 s difference, despite being statistically significant (*P* = 0.002), due to the high number of observations, appears physiologically negligible and reveals remarkable symmetry between the up- and down-phases of the sinusoids. Conversely, in CP_ex_, ${\dot{V}}_{{{\text{O}}}_{2}}$ *t*_*D* up_ was 36.1 ± 7.7 s and *t*_*D* down_ was 41.4 ± 9.7 s (*P* = 0.00001). The 5-s difference reveals remarkable asymmetry between the up- and down-phases of the sinusoids. Moreover, *t*_*D* up_ was 5.9 s longer in CP_ex_ than in CP-50_ex_, although the difference was not significant (*P* = 0.112); nevertheless, *t*_*D* down_ was 10.2 s and significantly (*P* = 0.009) longer in CP_ex_ than in CP-50_ex_. The same was the case for ${f}_{H}$.

### Blood lactate concentration, [La^−^], and rate of perceived exertion, RPE

As shown in Fig. 7, blood lactate concentration ([La^−^]) differed between the two protocols in all cycles, being always higher in CP_ex_ (*P* < 0.05) than in CP-50_ex_. In the former case, [La^−^] progressively increased with time, so that the third and the last cycle differed from the first (*P* = 0.022 and *P* = 0.0001, respectively). Conversely, in CP-50_ex_, [La^−^] was not different among the cycles, resulting in an average of 2.9 ± 0.3 mM. In addition, ${P}_{{{\text{ACO}}}_{2}}$ stayed stable (average: 38.9 ± 3.6 mmHg), independent of the cycle.

In almost all cycles, RPE_GEN_, RPE_MUSC_, and RPE_RESP_ were greater in CP_ex_ (*P* < 0.05) than in CP-50_ex_. In both CP-50_ex_ and CP_ex_, higher values of RPE_GEN_ (18.3 ± 0.3 and 18.4 ± 0.4 a.u., respectively), RPE_MUSC_ (8.3 ± 0.6 and 9.4 ± 0.4 a.u., respectively), and RPE_RESP_ (5.1 ± 0.4 and 6.6 ± 0.5 a.u., respectively) were registered in the last cycle compared to the first one (*P* < 0.001).

## Discussion

This study reports an overall analysis of the energy balance of sinusoidal work rates in two exercise domains, below and across the CP, taken as the upper limit of purely aerobic exercise. The results showed that i) the AMP of the ${\dot{V}}_{{{\text{O}}}_{2}}$ response to sinusoidal work was less than predicted from the corresponding expected difference in steady state ${\dot{V}}_{{{\text{O}}}_{2}}$, and the more so the higher the power at MP; ii) the sinusoids at CP_ex_ revealed asymmetries between the rising phase and the declining phase of the sinusoid, which was not the case for the sinusoids at CP-50_ex_; iii) the appearance of asymmetries is coherent with a greater role of anaerobic lactic metabolism to the energy balance; iv) no significant differences in *t*_*D*_*s* were observed between the protocols; v) contrary to CP-50_ex_, there was a clear tendency toward a positive drift of the ${\dot{V}}_{{{\text{O}}}_{2}}$ response during CP_ex_, associated with a progressive [La^−^] and RPE increase.

These results are only partially in agreement with the tested hypotheses. In fact, the hypothesized longer *t*_*D*_*s* at CP_ex_ than at CP-50_ex_ were observed only in *t*_*D* down_. As hypothesized, the tendency toward a drift in ${\dot{V}}_{{{\text{O}}}_{2}}$ was demonstrated in CP_ex_ only; however, such a drift appeared for ${\dot{V}}_{E}$ and ${f}_{H}$ in both protocols. Further, and in agreement with the tested hypothesis, a higher MP, lower AMP, and asymmetry between on- and off-phases of the sinusoids were observed during CP_ex_ with respect to CP-50_ex_.

${\dot{V}}_{{{\text{O}}}_{2}}$ MP remained steady for the entire CP-50_ex_ duration, thus suggesting that exercise intensity was sustained primarily by the aerobic metabolism (Poole et al. 2016; Ferretti et al. 2017, 2022). Considering that no differences appeared either in ${\dot{V}}_{{{\text{CO}}}_{2}}$ or in [La^−^] between the initial and the last cycles, the small rise in ${\dot{V}}_{E}$ MP might be associated with thermoregulatory effects. Indeed, since the rise in body temperature may lead to increased stimulation of central and/or peripheral chemoreceptors by H^+^, and of muscle metaboreceptors (Hayashi et al. 2006), the increase in ${\dot{V}}_{E}$ may contribute in keeping arterial blood pH and partial pressure of ${{\text{CO}}}_{2}$ unchanged. Nevertheless, the control mechanisms of ${\dot{V}}_{E}$ during exercise are complex. There is still an open debate about three main mechanisms thought to be involved in the control of breathing during exercise (central command, chemoreceptor stimulation and muscle mechanical and metabolic afferences) (Haouzi 2006; Forster et al. 2012), preventing to attain clear-cut conclusions on this issue.

In CP_ex_, other factors may have contributed to the increase in ${\dot{V}}_{{{\text{O}}}_{2}}$, ${\dot{V}}_{E}$, and ${f}_{H}$ MP in addition to the mechanisms discussed here above. Indeed, the growth of the cardiorespiratory and metabolic response could also be an effect of the slow component of ${\dot{V}}_{{{\text{O}}}_{2}}$, which is known to occur above the CP and which is mainly related to the recruitment of fast-twitch fibers and to a progressive shift toward fatty acid utilization during a long-lasting exercise (Jones et al. 2011; Ferretti 2015; Burnley and Jones 2018). The higher [La^−^] observed in our study in CP_ex_ seems to support this statement.

Contrary to what we hypothesized, although there are differences between *t*_*D* up_ and *t*_*D* down_ in CP_ex_, revealing asymmetries between up- and down-phase of the sinusoid, no significant differences in *t*_*D* up_ were found between CP_ex_ and CP-50_ex_ (Figs. 5 and 6). When measuring *t*_*D* up_ in CP-50_ex_, the MP is preceded by 2 min of exercise below the CP level, and therefore the slow component unlikely affects the dynamics of the ${\dot{{\text{V}}}}_{{{\text{O}}}_{2}}$ response, possibly leading to a shorter *t*_*D* up_ in CP-50_ex_ than in CP_ex_. Since, and likely for this reason, *t*_*D* down_ was 10.2 s and significantly longer in CP_ex_ than in CP-50_ex_, the lack of a significant differences in *t*_*D* up_ between the two sinusoidal exercise level may appear as a statistical artifice.

In CP-50_ex_, the entire sinusoid took place below the CP and below the MLSS (Beneke 2003; Jones et al. 2010; Leo et al. 2022; Ferretti et al. 2022). For a power AMP of 50 W, the mean ${\dot{{\text{V}}}}_{{{\text{O}}}_{2}}$ AMP was 488 ± 36 ml∙min^−1^ (see histogram of Fig. 4). During steady-state exercise on the cycle ergometer at 50 W, carried out at a pedalling frequency of 90–100 rpm (1.5 – 1.67 Hz), which we used as warm-up exercise, the net ${\dot{{\text{V}}}}_{{{\text{O}}}_{2}}$ was 775 ± 35 ml∙min^−1^. The corresponding gas exchange ratio at 50 W was 0.83 ± 0.02 and the mechanical efficiency was 0.20 ± 0.01, which is similar to the prediction made by di Prampero (2000).

The ${\dot{{\text{V}}}}_{{{\text{O}}}_{2}}$ AMP observed in the present study was lower than the measured steady-state ${\dot{{\text{V}}}}_{{{\text{O}}}_{2}}$ at 50 W (*P* < 0.001). This is another consequence of operating within a continuously oscillating exercise transient, as pointed out above, during which an oxygen deficit is built in the rising phase and an oxygen debt is paid in the declining phase of the sinusoid. Recently, Girardi et al. (2021a) conducted a theoretical analysis of the effects of *T* on ${\dot{{\text{V}}}}_{{{\text{O}}}_{2}}$ AMP. For *T* equal to 4 min, they predicted ${\dot{{\text{V}}}}_{{{\text{O}}}_{2}}$ AMP of some 70% of the ${\dot{{\text{V}}}}_{{{\text{O}}}_{2}}$ that is necessary to sustain a power corresponding to the mechanical AMP. This prediction is substantially supported by the present results, showing that the ${\dot{{\text{V}}}}_{{{\text{O}}}_{2}}$ AMP was 75% of the expected ${\dot{{\text{V}}}}_{{{\text{O}}}_{2}}$ for an equivalent steady state exercise at 50 W (mechanical AMP in the present study).

[La^−^] did not change from the first sinusoid on, indicating [La^−^] steady state and no further net lactate accumulation at each sinusoid. Therefore, the postulated oxygen deficit would be only alactic. Coherently, the sinusoids appear to be symmetrical, as long as *t*_*D* up_ was only 1 s lower than *t*_*D* down_.

In CP_ex_, the mechanical power MP was set at the CP, so that part of the cycle took place above it. A continuous rise in [La^−^] was found (Fig. 7), indicating a greater oxygen deficit than that incurring during CP-50_ex_. This is suggested also by the fact that the ${\dot{V}}_{{{\text{O}}}_{2}}$ AMP in CP_ex_ (304 ± 11 ml∙min^−1^) was less than in CP-50_ex_ (Fig. 4), even though *T* was the same. Since, in absence of [La^−^] accumulation during an exercise transient, the time constant of the ${\dot{V}}_{{{\text{O}}}_{2}}$ kinetics is invariant, power independent, and equal to that of phosphocreatine breakdown (di Prampero and Margaria 1968; Binzoni et al. 1997), and since the mechanical AMP was the same in CP_ex_ as in CP-50_ex_, the postulated higher oxygen deficit in the former than in the latter case can only be attributed to its anaerobic lactic component (facultative component of the oxygen deficit due to early lactate accumulation) (di Prampero 1981; Ferretti 2015).

On these premises, to shed light on these energetic issues, we tried to perform an analysis of the energy balance of the sinusoids, along the following lines. An estimate of the mechanical work above the nadir was done by computing the time integral of net power during a sinusoid, after subtracting the power at the nadir. Assuming a mechanical efficiency equal to that reported above, which, however, may carry along a slight overestimate of the actual exercise efficiency in CP_ex_ (Gaesser and Brooks 1975; Whipp and Wasserman 1969; Hesser et al. 1977), the corresponding metabolic energy consumption (sinusoid energy consumption, *E*_*s*_) was then computed. These values are reported in Table 2. Since we operate along a continuous sinusoidal change of power, implying continuous variations in oxygen deficit, *E*_*s*_ comprises energy deriving from aerobic and anaerobic energy sources. Thus, ${E}_{s,{{\text{O}}}_{2}}$ must be less than *E*_*s*_. ${E}_{s,{{\text{O}}}_{2}}$ was computed as the time integral of ${\dot{V}}_{{{\text{O}}}_{2}}$ during a sinusoid, after subtracting the ${\dot{V}}_{{{\text{O}}}_{2}}$ at the nadir. ${E}_{s,{{\text{O}}}_{2}}$ is also reported in Table 2.

Let us consider first CP-50_ex_: the sinusoidal variations in power (50 W above and below the MP) occurred entirely below the CP and the MLSS, and in fact from the first sinusoid on, no changes in [La^−^] and ${P}_{{{\text{ACO}}}_{2}}$ were observed. This means that no contributions from anaerobic lactic metabolism to the oxygen deficit occurred in this condition: thus, the difference between *E*_*s*_ and ${E}_{s,{{\text{O}}}_{2}}$, corresponding to the overall anaerobic energy contribution, turns equal to the alactic oxygen deficit built during a sinusoid (${E}_{s,{\text{Al}}}$). This is also reported in Table 2. Considering that a sinusoid is made by a rising part, in which the oxygen deficit is contracted, and a declining part, in which the oxygen debt is paid, the ${E}_{s,{\text{Al}}}$ reported in Table 2 is a net oxygen deficit (deficit contraction minus debt payment). Were contraction and payment of the alactic oxygen deficit characterized by the same time constant, ${E}_{s,{\text{Al}}}$ should turn out nil. However, ${E}_{s,{\text{Al}}}$ has a positive value, because the oxygen debt payment during recovery has a longer time constant than the oxygen deficit contraction during an on-transient (di Prampero 1981). This state of things would imply a progressive decline of muscle phosphocreatine concentration during CP-50_ex_, leading to exhaustion in a shorter time than during an equivalent constant-power exercise carried out at MP. This is coherent with progressive increase of RPE_MUSC_, and thus RPE_GEN_.

Let us now consider the CP_ex_ condition. In this case, part of the exercise would take place above CP, and thus above the MLSS. Therefore, a continuous increase in [La^−^] occurred. Thus, ${E}_{s,{{\text{O}}}_{2}}$ must be less than in CP-50_ex_, by an amount corresponding to the non-obligatory component of the oxygen deficit represented by anaerobic lactic metabolism during a sinusoid (${E}_{s,{\text{La}}}$). This can be computed as follows: in CP_ex_, the calculated net oxygen deficit is equal to ${E}_{s,{\text{Al}}}+{E}_{s,{\text{La}}}$, whereas in CP-50_ex_, it is equal to ${E}_{s,{\text{Al}}}$ only. Since the time constant of the obligatory component of the oxygen deficit is invariant and independent of power, and AMP is the same in both experimental conditions, as already pointed out, ${E}_{s,{\text{La}}}$ is given by the difference in oxygen deficit between CP_ex_ and CP-50_ex_. ${E}_{s,{\text{La}}}$ is also shown in Table 2. Assuming, from the first sinusoid on, a linear [La^−^] increase during CP_ex_, we calculated and reported in Table 2 also the estimated rate of [La^−^] accumulation during a sinusoid. This allowed an estimate of the energy equivalent of [La^−^] accumulation within an average sinusoid, as the ratio between ${E}_{s,{\text{La}}}$ and the [La^−^] accumulation within that sinusoid. This estimated value turned out equal to 2.73 ml·mM^−1^·kg^−1^, which is inside the range of values generally reported in the literature (see di Prampero 1981; di Prampero and Ferretti 1999; Ferretti 2015). Considering the potential sources of error in these estimates, a remarkable result indeed, even though, due to possibly lower efficiency of exercise in CP_ex_ than in CP-50_ex_, this value may turn out slightly overestimated.

**Table 2:** Energy balance components

	CP-50_ex_	CP_ex_
AMP ${\dot{V}}_{{{\text{O}}}_{2}}$ (ml∙min^−1^)	488 ± 36	304 ± 31
AMP* ${\dot{V}}_{{{\text{O}}}_{2}}$ (ml∙min^−1^)	775 ± 35	775 ± 35
${E}_{s}$ (ml∙O_2_)	2945 ± 149	2870 ± 170
${E}_{{\text{s}}, {{\text{O}}}_{2}}$ (ml∙O_2_)	2000 ± 125	1658 ± 126
${E}_{s}$ − ${\text{E}}_{{\text{s}}, {{\text{O}}}_{2}}$ (ml∙O_2_)	945 ± 187	1212 ± 246
${E}_{s,\mathrm{ La}}$ (ml∙O_2_)	0	392 ± 39
${E}_{s,\mathrm{ Al}}$ (ml∙O_2_)	945 ± 187	820 ± 260
Rate of [La^−^] accumulation in a sine (mM/cycle)	0	1.93 ± 0.20

Measured sinusoid amplitude of pulmonary oxygen uptake (AMP ${\dot{V}}_{{{\text{O}}}_{2}}$), theoretical sinusoid amplitude of pulmonary oxygen uptake (AMP* ${\dot{V}}_{{{\text{O}}}_{2}}$), sine energy consumption (*E*_*s*_), the amount of energy derived from aerobic source within the sine (${E}_{s,{{\text{O}}}_{2}}$), the alactic oxygen deficit built during a sine (${E}_{s,{\text{Al}}}$), anaerobic lactic metabolism (${E}_{s,{\text{La}}}$), rate of blood lactate ([La^−^]) accumulation in a sine in CP-50_ex_ and CP_ex_. Data are shown as mean ± standard error (SE). Note that ${E}_{s}$ − ${E}_{s, {{\text{O}}}_{2}}$ = ${E}_{s,{\text{Al}}}+{E}_{s,{\text{La}}}$. AMP* ${\dot{V}}_{{{\text{O}}}_{2}}$ was calculated during steady state exercise on the cycle ergometer at 50 W, carried out at a pedalling frequency of 90–100 rpm (1.5–1.67 Hz), which we used as warm-up exercise

In CP-50_ex_, the MP for mechanical power was 168 ± 13 W. If the phase I of the ${\dot{V}}_{{{\text{O}}}_{2}}$ response upon exercise start is due to sudden vagal withdrawal (Lador et al. 2006; Fontolliet et al. 2021), one should expect no rapid phase I when the investigated power increase is imposed from the ongoing MP. This being the case, in CP-50_ex_, we may assume that the metabolic response to exercise corresponds to that of a first-order system (Whipp et al. 1982; Haouzi et al. 1993; Ferretti 2023). This assumption has not been tested so far. However, the lack of [La^−^] accumulation, apart from early lactate accumulation (Cerretelli et al. 1979), and the stability of ${P}_{{{\text{ACO}}}_{2}}$ demonstrate that the experiments were carried out entirely in the fully aerobic domain (Ferretti et al. 2017, 2022). Moreover, we found a substantial symmetry between the up- and down-phases of the sinusoids. Both these observations are in line with the assumption of a first-order system.

To detect the presence of fatigue, we investigated the cardiorespiratory and metabolic responses to sinusoidal exercises up to exhaustion. To this purpose, we had to apply a cycle-by-cycle analysis of the investigated variables. Previous studies chose to overlap all the cycles performed to strengthen the analysis of the physiological response to sinusoidal exercise (Wigertz 1970; Casaburi et al. 1977; Bakker et al. 1980; Fukuoka and Ikegami 1990; Haouzi et al. 1993; Fukuoka et al. 1995, 1997; Wells et al. 2007; Nicolò et al. 2018; Girardi et al. 2021b). In those studies, the response to sinusoidal work rate was investigated only in protocols of relatively short duration. However, in the present protocols carried out up to exhaustion, significant differences among successive cycles were found for most of the investigated variables. This undermined the possible use of the overlapping procedure in this study.

The progressive increase in ${f}_{H}$ MP, in accordance with Haouzi et al. (1993), during both protocols may be explained by a gradual hyper-activation of the sympathetic branch of the autonomic nervous system (Orizio et al. 1988; Rowell and O’Leary 1990), a rise in body’s internal temperature (Rowell and O’Leary 1990; González-Alonso et al. 1997), an increase in blood catecholamine concentration (Orizio et al. 1988; Urhausen et al. 1994), and/or a gradual impairment of hydration status (González-Alonso et al. 1997). This last, together with skin vasodilation, might concur to a venous return reduction and, consequently, to a pre-load decrease that might have driven the progressive rise in ${f}_{H}$ MP (Lonsdorfer-Wolf et al. 2003). However, if the stroke volume remains constant during both sinusoidal exercises, which might be the case indeed in the power range used in this study, the hypothesis of a reduction in cardiac pre-load might have to be rejected. The patterns of the stroke volume changes during sinusoidal exercise were not investigated so far, to the best of our knowledge, and should be the object of future studies.

## Conclusion

This study represents an attempt at analyzing the energetics of sinusoidal exercise below and across CP and MLSS. A substantial energetic compatibility of the data with current theories of the energetics of muscular exercise appeared. The progressively lower aerobic component during CP_ex_ than during CP-50_ex_ is associated with lactate accumulation in the former condition. This is coherent with the observed greater asymmetries and lower AMP for ${\dot{V}}_{{{\text{O}}}_{2}}$ in the CP_ex_ sinusoids than in the CP-50_ex_ sinusoids. The asymmetries observed in CP_ex_ suggest a progressive decline of muscle phosphocreatine concentration with time, implying the onset of fatigue. This may explain, at least in part, why, in athletic competitions, better performances are obtained when running at constant speed on flat terrain than when running at varying speed on undulating terrain.

### Supplementary Information

Below is the link to the electronic supplementary material.

Supplementary file1 (DOCX 750 KB)

## Data Availability

Data generated during and/or analyzed during the current study are available as Supporting Information.

## Abbreviations

AMP: Sinusoid amplitude
CP: Critical power
CP-50_ex_: Sinusoidal protocol having the MP for power set 50 W below the CP
CP_ex_: Sinusoidal protocol having the MP for power set at the CP
*E*_*s*_: Sinusoid energy consumption
${E}_{s,{\text{Al}}}$: Alactic oxygen deficit built during a sinusoid
${E}_{s,{\text{La}}}$: Anaerobic lactic metabolism during a sinusoid
${E}_{s,{{\text{O}}}_{2}}$: Amount of energy from aerobic sources during a sinusoid
$${f}_{H}$$: Heart rate
$${f}_{R}$$: Respiratory rate
[La^−^]: Blood lactate concentration
MLSS: Maximal lactate steady state
MP: Sinusoid midpoint
${P}_{{{\text{ACO}}}_{2}}$: Alveolar carbon dioxide pressure
RPE_GEN_: Rate of general perceived exertion
RPE_MUSC_: Rate of muscular perceived exertion
RPE_RESP_: Rate of respiratory perceived exertion
T: Sinusoid period
*t*_D_: Time delay
*t*_D down_: Time delay at downward midpoint crossing
*t*_D up_: Time delay at upward midpoint crossing
${\dot{V}}_{{{\text{CO}}}_{2}}$: Pulmonary carbon dioxide output
$${\dot{V}}_{E}$$: Expiratory ventilation
${\dot{V}}_{{{\text{O}}}_{2}}$: Pulmonary oxygen uptake
${\dot{V}}_{{{\text{O}}}_{2}{\text{max}}}$: Maximum oxygen uptake
*V*_*T*_: Tidal volume
$$\dot{W}$$: Mechanical power
${\dot{W}}_{{\text{max}}}$: Maximal mechanical aerobic power
