# Supplementary material for: Energetics of sinusoidal exercise below and across critical power and the effects of fatigue
Source: Eur J Appl Physiol. 2024 Jan 19;124(6):1845–59. doi: 10.1007/s00421-023-05410-1 (PMC11130025; doi:10.1007/s00421-023-05410-1)
Supplement: Supplementary file 1 — Supplementary file1 (DOCX 750 KB) [file 421_2023_5410_MOESM1_ESM.docx]

**Running title: Energetics of sinusoidal exercise**

**Energetics of sinusoidal exercise below and across critical power and the effects of fatigue.**

Marta Borrelli^1^, Sheida Shokohyar^1^, Susanna Rampichini^1*^, Paolo Bruseghini^2^, Christian Doria^1^, Eloisa Guglielmina Limonta^1,3^, Guido Ferretti^2^, Fabio Esposito^1,3^.

^1^Department of Biomedical Sciences for Health, Università degli Studi di Milano, Italia

^2^ Department of Molecular and Translational Medicine, University of Brescia, Italia

^3^ IRCCS Ospedale Galeazzi - Sant’Ambrogio, Via Cristina Belgioioso, 173, 20157, Milan, Italy

* Corresponding author

**Correspondance:**

Susanna Rampichini

Via Giuseppe Colombo 71,

20133 Milan, Italy

Email: susanna.rampichini@unimi.it

Phone: (+39) 02 503 14640

**
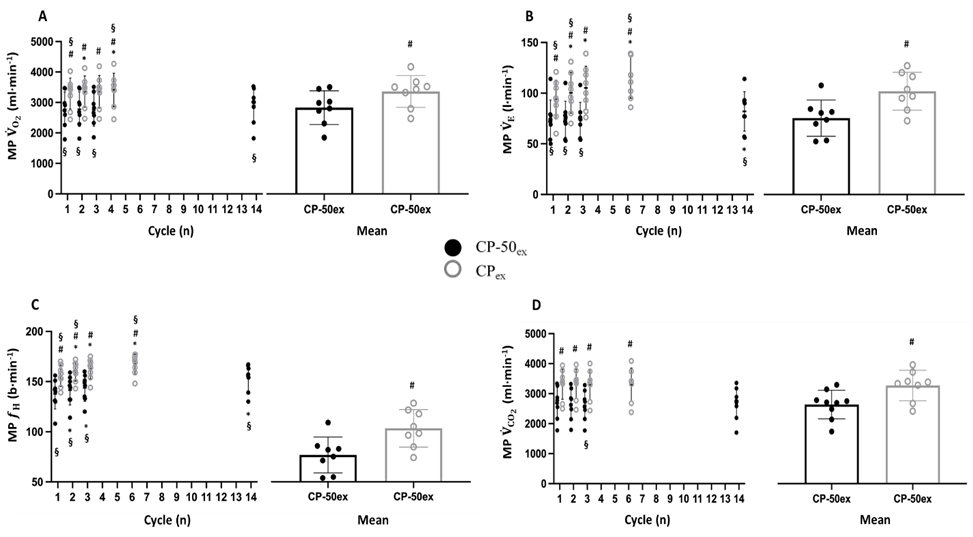
Figure 1:** Midpoint (MP) response of pulmonary oxygen uptake, $\dot{V}_{O_{2}}$ (panel A), ventilation, $\dot{V}_{E}$ (panel B), heart rate, $f_{H}$ (panel C), carbon dioxide production, $\dot{V}_{{CO}_{2}}$ (panel D) to each sinewave (sin) of CP_ex_ (grey circles) and CP-50_ex_ (black circles). The column charts represent the average of all cycles during CP_ex_ (grey bars) and CP-50_ex_ (black bars). *p<0.05 vs sine 1; ^#^ p<0.05 vs CP_ex_; ^§^p<0.05 vs overlapped sines. Data are shown as mean ± standard deviation (SD)

**Figure 2:** Amplitude (AMP) response between midpoint and zenith of pulmonary oxygen uptake, $\dot{V}_{O_{2}}$ (panel a), ventilation, $\dot{V}_{E}$ (panel b), heart rate, $f_{H}$ (panel c), carbon dioxide production, $\dot{V}_{{CO}_{2}}$ (panel d) to each sinewave (sin) of CP_ex_ (grey circles) and CP-50_ex_ (black circles). The column charts represent the average of all cycles during CP_ex_ (grey bars) and CP-50_ex_ (black bars). *p<0.05 vs sine 1; ^#^ p<0.05 vs CP_ex_; ^§^p<0.05 vs overlapped sines. Data are shown as mean ± standard deviation (SD)


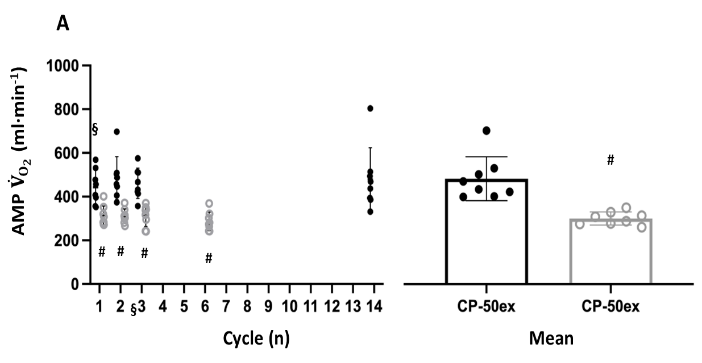

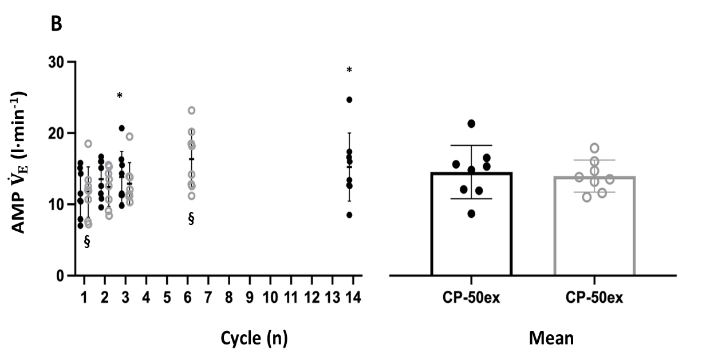

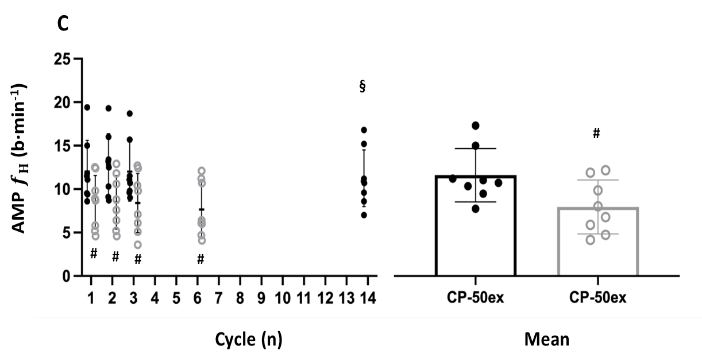

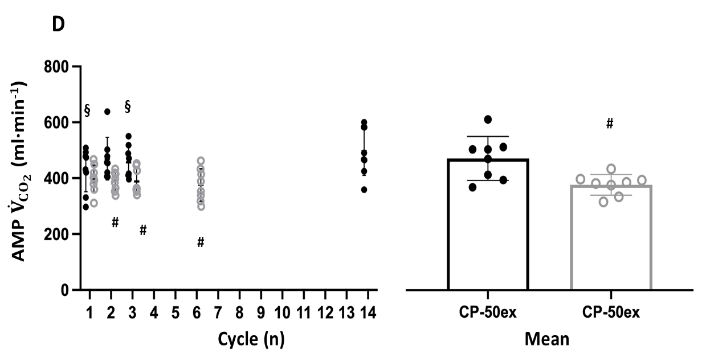

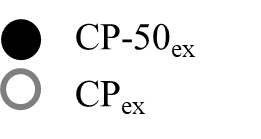


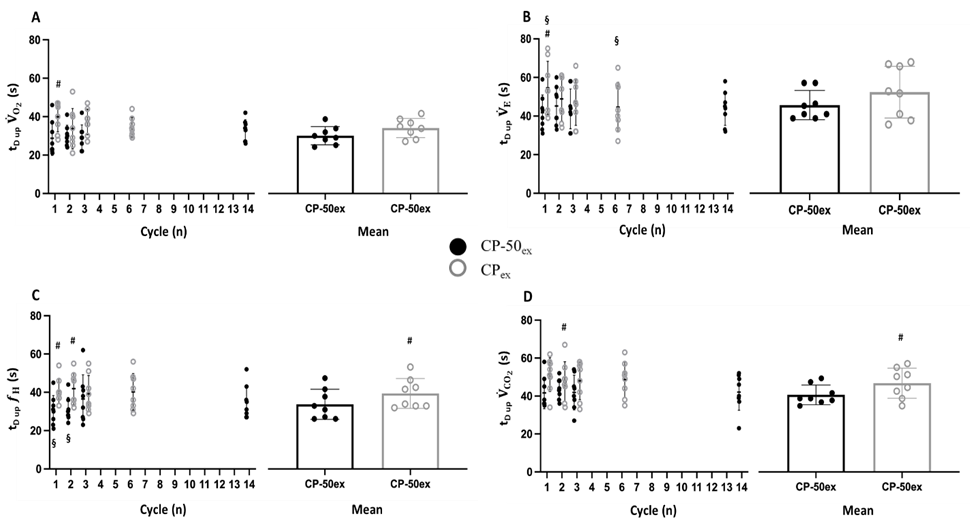
**Figure 3:** Time delay upward MP crossing (t_D up_) of pulmonary oxygen uptake, $\dot{V}_{O_{2}}$ (panel A), ventilation, $\dot{V}_{E}$ (panel B), heart rate, $f_{H}$ (panel C), carbon dioxide production, $\dot{V}_{{CO}_{2}}$ (panel D) to each sinewave (sin) of CP_ex_ (grey circles) and CP-50_ex_ (black circles). The column charts represent the average of all cycles during CP_ex_ (grey bars) and CP-50_ex_ (black bars). *p<0.05 vs sine 1; ^#^ p<0.05 vs CP_ex_; ^§^p<0.05 vs overlapped sines. Data are shown as mean ± standard deviation (SD)

**
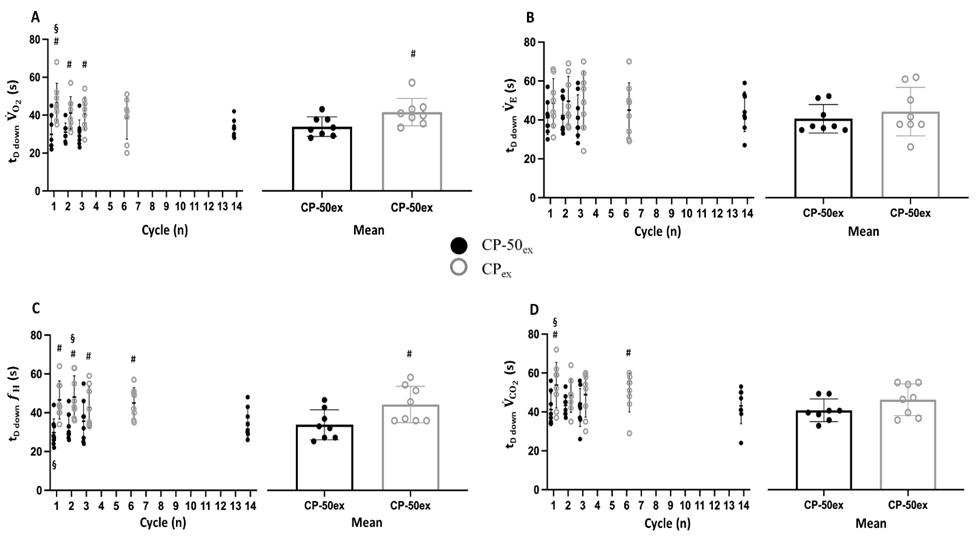
Figure 4:** Time delay downward MP crossing (t_D down_) of pulmonary oxygen uptake, $\dot{V}_{O_{2}}$ (panel A), ventilation, $\dot{V}_{E}$ (panel B), heart rate, $f_{H}$ (panel C), carbon dioxide production, $\dot{V}_{{CO}_{2}}$ (panel D) to each sinewave (sin) of CP_ex_ (grey circles) and CP-50_ex_ (black circles). The column charts represent the average of all cycles during CP_ex_ (grey bars) and CP-50_ex_ (black bars). *p<0.05 vs sine 1; ^#^ p<0.05 vs CP_ex_; ^§^p<0.05 vs overlapped sines. Data are shown as mean ± standard deviation (SD)


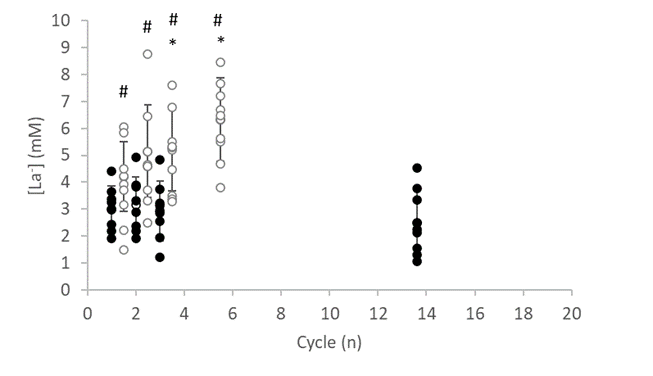
**Figure 7** Blood lactate concentration ([La^-^]) to each cycle of CP_ex_ (grey circles) and CP-50_ex_ (black circles). * p<0.05 vs sine 1; ^#^ p<0.05 vs CP_ex_. Data are shown as mean ± standard deviation (SD)

**Table 1:** Within protocols effect in midpoint (MP), amplitude (AMP) and time delay upward and downward MP crossing (t_D up_ and t_D down_, respectively) response of pulmonary oxygen uptake ($\dot{V}_{O_{2}}$), ventilation ($\dot{V}_{E}$), carbon dioxide production ($\dot{V}_{\mathrm{CO}_{2}}$) and heart rate ($f_{H}$) to each sinewave (sin) and to average sines of CP-50_ex_ (table 1a) and CP_ex_ (table 1b).

Data are shown as mean ± standard error (SE). *p<0.05 vs sine 1

| **Table 1a** | **CP-50_ex_** | | | |
| --- | --- | --- | --- | --- |
|  | **Sin1** | **Sin2** | **Sin3** | **SinLast** |
|  |  |  |  |  |
| **MP** ${\dot{\mathbf{V}}}_{\mathbf{O}_{\mathbf{2}}}$ **(ml∙min^-1^)** | 2776 ± 202 | 2783 ± 201 | 2758 ± 189 | 2900 ± 202 |
| **MP** ${\dot{\mathbf{V}}}_{\mathbf{E}}$ **(l∙min^-1^)** | 74 ± 7 | 74 ± 6 | 74 ± 6 | 82 ± 7*  P = 0.042  g = 1.05  95% CI = -0.0004 - 2.09 |
| **MP** ${\dot{\mathbf{V}}}_{\mathbf{C}\mathbf{O}_{\mathbf{2}}}$ **(ml∙min^-1^)** | 2681 ± 185 | 2652 ± 180 | 2611 ± 174 | 2675 ± 187 |
| **MP** $\boldsymbol{f}_{\mathbf{H}}$ **(b∙min^-1^)** | 138 ± 5 | 141 ± 5*  P = 0.009  g = 0.23  95% CI = -0.75 - 1.22 | 144 ± 5*  P = 0.004  g = 0.40  95% CI = -0.59 – 1.39 | 154 ± 5*  P = 0.002  g =1.10  95% CI = 0.05 - 2.15 |
|  |  |  |  |  |
| **AMP** ${\dot{\mathbf{V}}}_{\mathbf{O}_{\mathbf{2}}}$ **(ml∙min^-1^)** | 443 ± 28 | 485 ± 35 | 461 ± 24 | 479 ± 51 |
| **AMP** ${\dot{\mathbf{V}}}_{\mathbf{E}}$ **(l∙min^-1^)** | 11.6 ± 1.1 | 13.5 ± 1.0 | 13.8 ± 1.3*  P = 0.05  g = 0.60  95% CI = -0.40 – 1.60 | 15.3 ± 1.7*  P = 0.04  g = 0.84  95% CI = -0-18 – 1.86 |
| **AMP** ${\dot{\mathbf{V}}}_{\mathbf{C}\mathbf{O}_{\mathbf{2}}}$ **(ml∙min^-1^)** | 429 ± 27 | 470 ± 27 | 457 ± 20 | 493 ± 30 |
| **AMP** $\boldsymbol{f}_{\mathbf{H}}$ **(b∙min^-1^)** | 12.0 ± 1.3 | 12.8 ± 1.3 | 12.0 ± 1.2 | 11.2 ± 1.2 |
|  |  |  |  |  |
| **t_D up_** ${\dot{\mathbf{V}}}_{\mathbf{O}_{\mathbf{2}}}$ **(s)** | 28.8 ± 3.1 | 30.1 ± 1.9 | 29.3 ± 2.3 | 32.8 ± 2.0 |
| **t_D up_** ${\dot{\mathbf{V}}}_{\mathbf{E}}$ **(s)** | 41.8 ± 3.2 | 45.3 ± 3.5 | 43.8 ± 3.6 | 44.0 ± 3.1 |
| **t_D up_** ${\dot{\mathbf{V}}}_{\mathbf{C}\mathbf{O}_{\mathbf{2}}}$ **(s)** | 41.6 ± 2.9 | 43.8 ± 1.9 | 41.9 ± 3.2 | 42.1 ± 3.4 |
| **t_D up_** $\boldsymbol{f}_{\mathbf{H}}$ **(s)** | 30.6 ± 2.7 | 31.0 ± 2.2 | 36.5 ± 4.5 | 35.0 ± 3.1 |
|  |  |  |  |  |
| **t_D down_** ${\dot{\mathbf{V}}}_{\mathbf{O}_{\mathbf{2}}}$ **(s)** | 29.9 ± 3.2 | 31.0 ± 1.7 | 30.8 ± 2.4 | 33.3 ± 1.6 |
| **t_D down_** ${\dot{\mathbf{V}}}_{\mathbf{E}}$ **(s)** | 41.1 ± 3.1 | 42.3 ± 3.2 | 41.8 ± 3.9 | 44.3 ± 3.6 |
| **t_D down_** ${\dot{\mathbf{V}}}_{\mathbf{C}\mathbf{O}_{\mathbf{2}}}$ **(s)** | 41.3 ± 3.0 | 43.4 ± 1.9 | 42.3 ± 3.4 | 43.1 ± 3.3 |
| **t_D down_** $\boldsymbol{f}_{\mathbf{H}}$ **(s)** | 29.8 ± 2.5 | 32.4 ± 2.4 | 35.6 ± 3.8 | 34.9 ± 2.7 |
|  |  |  |  |  |
| **Table 1b** | **CP_ex_** | | | |
|  | **Sin1** | **Sin2** | **Sin3** | **SinLast** |
|  |  |  |  |  |
| **MP** ${\dot{\mathbf{V}}}_{\mathbf{O}_{\mathbf{2}}}$ **(ml∙min^-1^)** | 3293 ± 180 | 3359 ± 182*  P = 0.038  g = 0.12  95% CI = -0.86 – 1.10 | 3354 ± 189 | 3410 ± 194*  P = 0.05  g = 0.21  95% CI = -0.78 – 1.19 |
| **MP** ${\dot{\mathbf{V}}}_{\mathbf{E}}$ **(l∙min^-1^)** | 94 ± 7 | 100 ± 7*  P = 0.005  g = 0.32  95% CI = -0.67 – 1.30 | 105 ± 8*  P = 0.003  g = 0.53  95% CI = -0.47 – 1.53 | 116 ± 7*  P <0.001  g = 1.05  95% CI = 0.0004 – 2.09 |
| **MP** ${\dot{\mathbf{V}}}_{\mathbf{C}\mathbf{O}_{\mathbf{2}}}$ **(ml∙min^-1^)** | 3318 ± 177 | 3325 ± 174 | 3301 ± 179 | 3308 ± 193 |
| **MP** $\boldsymbol{f}_{\mathbf{H}}$ **(b∙min^-1^)** | 156 ± 4 | 160 ± 4*  P = 0.010  g = 0.41  95% CI = -0.58- 1.40 | 163 ± 4*  P = 0.002  g = 0.64  95% CI = -0.37 – 1.64 | 167 ± 4*  P = 0.003  g = 1.07  95% CI = 0.02 – 2.12 |
|  |  |  |  |  |
| **AMP** ${\dot{\mathbf{V}}}_{\mathbf{O}_{\mathbf{2}}}$ **(ml∙min^-1^)** | 311 ± 16 | 311 ± 12 | 312 ± 17 | 287 ± 15 |
| **AMP** ${\dot{\mathbf{V}}}_{\mathbf{E}}$ **(l∙min^-1^)** | 11.7 ± 1.3 | 12.4 ± 1.0 | 12.9 ± 1.0 | 16.4 ± 1.5 |
| **AMP** ${\dot{\mathbf{V}}}_{\mathbf{C}\mathbf{O}_{\mathbf{2}}}$ **(ml∙min^-1^)** | 398 ± 18 | 388 ± 12 | 388 ± 16 | 376 ± 21 |
| **AMP** $\boldsymbol{f}_{\mathbf{H}}$ **(b∙min^-1^)** | 8.5 ±1.1 | 8.5 ± 1.1 | 8.4 ± 1.2 | 7.7 ± 1.1 |
|  |  |  |  |  |
| **t_D up_** ${\dot{\mathbf{V}}}_{\mathbf{O}_{\mathbf{2}}}$ **(s)** | 39.6 ± 2.7 | 33.5 ± 3.7 | 37.0 ± 2.3 | 34.2 ± 1.8 |
| **t_D up_** ${\dot{\mathbf{V}}}_{\mathbf{E}}$ **(s)** | 54.2 ± 4.8 | 48.6 ± 4.0 | 47.1 ± 4.3 | 44.0 ± 4.6 |
| **t_D up_** ${\dot{\mathbf{V}}}_{\mathbf{C}\mathbf{O}_{\mathbf{2}}}$ **(s)** | 50.9 ± 3.1 | 47.4 ± 3.7 | 47.8 ± 3.5 | 48.4 ± 3.3 |
| **t_D up_** $\boldsymbol{f}_{\mathbf{H}}$ **(s)** | 40.4 ± 2.3 | 41.7 ± 3.0 | 39.0 ± 3.4 | 39.9 ± 3.4 |
|  |  |  |  |  |
| **t_D down_** ${\dot{\mathbf{V}}}_{\mathbf{O}_{\mathbf{2}}}$ **(s)** | 46.1 ± 3.6 | 41.1 ± 3.0 | 40.5 ± 3.2 | 38.1 ± 3.9 |
| **t_D down_** ${\dot{\mathbf{V}}}_{\mathbf{E}}$ **(s)** | 48.3 ± 4.5 | 49.4 ± 4.5 | 49.3 ± 5.1 | 44.6 ± 5.1 |
| **t_D down_** ${\dot{\mathbf{V}}}_{\mathbf{C}\mathbf{O}_{\mathbf{2}}}$ **(s)** | 53.4 ± 4.1 | 48.1 ± 3.1 | 48.6 ± 4.1 | 49.6 ± 3.6 |
| **t_D down_** $\boldsymbol{f}_{\mathbf{H}}$ **(s)** | 46.4 ± 3.5 | 47.9 ± 3.8 | 42.7 ± 3.8  P=0.047  t=2.797  g=0.34 | 44.6 ± 2.8 |

**Table 2:** Within protocols effect in blood lactate concentration ([La^-^]), rates of perceived exertion on a general (RPE_GEN_; Borg 6-20), respiratory and muscular (RPE_MUSC_ and RPE_RESP_; CR-10) values of each sinewave (sin) of CP-50_ex_ (table 2a) and CP_ex_ (table 2b).

Data are shown as mean ± standard error (SE). *p<0.05 vs sine 1.

| **Table 2a** | **CP-50_ex_** | | | |
| --- | --- | --- | --- | --- |
|  | **Sin1** | **Sin2** | **Sin3** | **SinLast** |
|  |  |  |  |  |
| **[La^-^] (mM)** | 3.0 ± 0.3 | 3.2 ± 0.4 | 2.9 ± 0.4 | 2.5 ± 0.4 |
| **RPE_GEN 6-20_ (u.a.)** | 12.0 ± 0.6 | 12.9 ± 0.4 | 13.1 ± 0.4 | 18.3 ± 0.3^*^  P = 0.001  g = 4.38  CI_95%_ = 2.57 – 6.18 |
| **RPE_MUS 0-10_ (u.a.)** | 3.3 ± 0.2 | 3.9 ± 0.3 | 4.1 ± 0.3 | 8.3 ± 0.6^*^  P <0.001  g = 3.91  CI_95%_ = 2.24 – 5.58 |
| **RPE_RESP_ _0-10_ (u.a.)** | 2.6 ± 0.6 | 2.6 ± 0.5 | 3.0 ± 0.5 | 5.1 ± 0.4^*^  P = 0.010  g = 1.74  CI_95%_ = 0.59 – 2.89 |

| **Table 2b** | **CP_ex_**  **_ex_** | | | |
| --- | --- | --- | --- | --- |
|  | **Sin1** | **Sin2** | **Sin3** | **SinLast** |
|  |  |  |  |  |
| **[La^-^] (mM)** | 4.2 ± 0.5^#^ | 5.2 ± 0.6 | 5.2 ± 0.5^*^  P = 0.022  g = 0.67  CI_95%_ = -0.34 – 1.68 | 6.3 ± 0.5^*^  P <0.001  g = 1.41  CI_95%_ = 0.31 – 2.50 |
| **RPE_GEN 6-20_ (u.a.)** | 13.6 ± 0.5 | 15.0 ± 0.7^*;#^  P = 0.023  g = 0.73  CI_95%_ = -0.28 – 1.75 | 15.4 ± 0.6^*^  P = 0.001  g = 1.01  CI_95%_ = -0.03 – 2.05 | 18.4 ± 0.4^*^  P <0.001  g = 3.45  CI_95%_ = 1.90 – 4.99 |
| **RPE_MUS 0-10_ (u.a.)** | 5.0 ± 0.4 | 5.9 ± 0.6 | 6.8 ± 0.6^*^  P = 0.037  g = 1.14  CI_95%_ = 0.08 – 2.20 | 9.4 ± 0.4^*^  P <0.001  g = 3.66  CI_95%_ = 2.06 – 5.26 |
| **RPE_RESP_ _0-10_ (u.a.)** | 3.2 ± 0.3 | 3.9 ± 0.5^#^ | 4.8 ± 0.5^*^  P = 0.05  g = 3.20  CI_95%_ = 1.72 – 4.68 | 6.6 ± 0.5^*^  P = 0.001  g = 4.66  CI_95%_ = 2.77 – 6.55 |

**Table 3:** Between protocols effect in midpoint (MP), amplitude (AMP) and time delay upward and downward MP crossing (t_D up_ and t_D down_, respectively) response of pulmonary oxygen uptake ($\dot{V}_{O_{2}}$), ventilation ($\dot{V}_{E}$), carbon dioxide production ($\dot{V}_{{CO}_{2}}$) and heart rate ($f_{H}$) to each sinewave (sin) and to average sines of CP-50_ex_ and CP_ex_. Data are shown as mean ± standard error (SE). ^#^ p<0.05 vs CP-50_ex_

| **Parameter** | **CP-50_ex_** | | | | |  | **CP_ex_** | | | | |
| --- | --- | --- | --- | --- | --- | --- | --- | --- | --- | --- | --- |
|  | **Average** | **Sin1** | **Sin2** | **Sin3** | **SinLast** |  | **Average** | **Sin1** | **Sin2** | **Sin3** | **SinLast** |
|  |  |  |  |  |  |  |  |  |  |  |  |
| **MP** ${\dot{\mathbf{V}}}_{\mathbf{O}_{\mathbf{2}}}$ **(ml∙min^-1^)** | 2826 ± 195 | 2776 ± 202 | 2783 ± 201 | 2758 ± 189 | 2900 ± 202 |  | 3355 ± 185^#^  t = 10.673  P <0.001  g = 0.909  CI_95%_ = 0.30 – 1.52 | 3293 ± 180^#^  P <0.001  g = 0.90  CI_95%_ = -012 – 1.93 | 3359 ± 182^#^  P <0.001  g = 1.01  CI_95%_ = -0.03 – 2.05 | 3354 ± 189^#^  P <0.001  g = 1.05  CI_95%_ = 0.16 – 1.89 | 3410 ± 194^#^  P <0.001  g = 0.86  CI_95%_ = -0.01 – 2.10 |
| **MP** ${\dot{\mathbf{V}}}_{\mathbf{E}}$ **(l∙min^-1^)** | 78 ± 6 | 74 ± 7 | 74 ± 6 | 74 ± 6 | 82 ± 7 |  | 105 ± 7^#^  t = 9.489  P <0.001  g = 1.374  CI_95%_ = 0.44 – 2.31 | 94 ± 7^#^  P = 0.001  g = 0.98  CI_95%_ = -0.05 – 2.02 | 100 ± 7^#^  P <0.001  g = 1.29  CI_95%_ = 0.22 – 2.37 | 105 ± 8^#^  P <0.001  g = 1.53  CI_95%_ = 0.41 – 2.64 | 116 ± 7^#^  P <0.001  g = 1.59  CI_95%_ = 0.46 – 2.71 |
| **MP** ${\dot{\mathbf{V}}}_{\mathbf{C}\mathbf{O}_{\mathbf{2}}}$ **(ml∙min^-1^)** | 2661 ± 182 | 2681 ± 185 | 2652 ± 180 | 2611 ± 174 | 2675 ± 187 |  | 3309 ± 182^#^  t = 21.144  P <0.001  g = 1.193  CI_95%_ = 0.43 – 1.96 | 3318 ± 177^#^  P <0.001  g = 1.17  CI_95%_ = 0.11 – 1.27 | 3325 ± 174^#^  P <0.001  g = 1.27  CI_95%_ = 0.20 – 2.35 | 3301 ± 179^#^  P <0.001  g = 1.31  CI_95%_ = 0.23 – 2.39 | 3308 ± 193^#^  P <0.001  g = 1.11  CI_95%_ = 0.06 – 2.17 |
| **MP** $\boldsymbol{f}_{\mathbf{H}}$ **(b∙min^-1^)** | 147 ± 5 | 138 ± 5 | 141 ± 5 | 144 ± 5 | 154 ± 5 |  | 162 ± 4^#^  t = 4.242  P = 0.004  g = 1.207  CI_95%_ = 0.19 – 2.22 | 156 ± 4^#^  P = 0.002  g = 1.33  CI_95%_ = 0.25 – 2.41 | 160 ± 4^#^  P = 0.001  g = 1.43  CI_95%_ = 0.33 – 2.53 | 163 ± 4^#^  P = 0.001  g = 1.52  CI_95%_ = 0.41 – 2.63 | 167 ± 4^#^  P = 0.011  g = 1.09  CI_95%_ = 0.04 – 2.14 |
|  |  |  |  |  |  |  |  |  |  |  |  |
| **AMP** ${\dot{\mathbf{V}}}_{\mathbf{O}_{\mathbf{2}}}$ **(ml∙min^-1^)** | 488 ± 36 | 443 ± 28 | 485 ± 35 | 461 ± 24 | 479 ± 51 |  | 304 ± 11^#^  t = 4.251  P = 0.004  g = 2.557  CI_95%_ = 0.40 – 4.47 | 311 ± 16^#^  P = 0.011  g = 1.92  CI_95%_ = 0.74 – 3.10 | 311 ± 12^#^  P = 0.004  g = 2.25  CI_95%_ = 1.00 – 3.50 | 312 ± 17^#^  P = 0.003  g = 2.36  CI_95%_ = 1.09 – 3.64 | 287 ± 15^#^  P = 0.012  g = 1.70  CI_95%_ = 0.55 – 2.84 |
| **AMP** ${\dot{\mathbf{V}}}_{\mathbf{E}}$ **(l∙min^-1^)** | 14.5 ± 1.3 | 11.6 ± 1.1 | 13.5 ± 1.0 | 13.8 ± 1.3 | 15.3 ± 1.7 |  | 14.0 ± 0.8 | 11.7 ± 1.3 | 12.4 ± 1.0 | 12.9 ± 1.0 | 16.4 ± 1.5 |
| **AMP** ${\dot{\mathbf{V}}}_{\mathbf{C}\mathbf{O}_{\mathbf{2}}}$ **(ml∙min^-1^)** | 487 ± 29 | 429 ± 27 | 470 ± 27 | 457 ± 20 | 493 ± 30 |  | 389 ± 13^#^  t = 2.579  P = 0.037  g = 1.520  CI_95%_ = -0.17 – 3.21 | 398 ± 18 | 388 ± 12^#^  P = 0.05  g = 1.31  CI_95%_ = 0.23 – 2.39 | 388 ± 168^#^  P = 0.05  g = 1.25  CI_95%_ = 0.18 – 2.33 | 376 ± 21^#^  P = 0.03  g = 1.56  CI_95%_ = 0.44 – 2.67 |
| **AMP** $\boldsymbol{f}_{\mathbf{H}}$ **(b∙min^-1^)** | 12.0 ± 1.1 | 12.0 ± 1.3 | 12.8 ± 1.3 | 12.0 ± 1.2 | 11.2 ± 1.2 |  | 8.2 ±1.1^#^  t = 7.446  P <0.001  g = 1.121  CI_95%_ = 0.33 – 1.91 | 8.5 ±1.1^#^  P = 0.003  g = 0.46  CI_95%_ = -0.54 – 1.45 | 8.5 ± 1.1^#^  P = 0.001  g = 1.31  CI_95%_ = 0.23 – 2.39 | 8.4 ± 1.2^#^  P = 0.001  g = 1.25  CI_95%_ = 0.18 – 2.33 | 7.7 ± 1.1^#^  P = 0.001  g = 1.56  CI_95%_ = 0.44 – 2.67 |
|  |  |  |  |  |  |  |  |  |  |  |  |
| **t_D up_** ${\dot{\mathbf{V}}}_{\mathbf{O}_{\mathbf{2}}}$ **(s)** | 31.0 ± 1.7 | 28.8 ± 3.1 | 30.1 ± 1.9 | 29.3 ± 2.3 | 32.8 ± 2.0 |  | 35.1 ± 1.8 | 39.6 ± 2.7^#^  P = 0.016  g = 1.24  CI_95%_ = 0.17 – 2.31 | 33.5 ± 3.7 | 37.0 ± 2.3 | 34.2 ± 1.8 |
| **t_D up_** ${\dot{\mathbf{V}}}_{\mathbf{E}}$ **(s)** | 42.5 ± 2.5 | 41.8 ± 3.2 | 45.3 ± 3.5 | 43.8 ± 3.6 | 44.0 ± 3.1 |  | 48.8 ± 4.4 | 54.2 ± 4.8^#^  P = 0.05  g = 1.01  CI_95%_ = -0.03 – 2.05 | 48.6 ± 4.0 | 47.1 ± 4.3 | 44.0 ± 4.6 |
| **t_D up_** ${\dot{\mathbf{V}}}_{\mathbf{C}\mathbf{O}_{\mathbf{2}}}$ **(s)** | 42.0 ± 1.9 | 41.6 ± 2.9 | 43.8 ± 1.9 | 41.9±3.2 | 42.1±3.4 |  | 48.2 ± 2.9^#^  P = 0.063  t = 2.206  g = 0.83  CI_95%_ = -0.19 – 1.86 | 50.9 ± 3.1^#^  P = 0.021  g = 1.03  CI_95%_ = -0.01 – 2.08 | 47.4 ± 3.7 | 47.8 ± 3.5 | 48.4 ± 3.3 |
| **t_D up_** $\boldsymbol{f}_{\mathbf{H}}$ **(s)** | 34.7 ± 2.9 | 30.6 ± 2.7 | 31.0 ± 2.2 | 36.5 ± 4.5 | 35.0 ± 3.1 |  | 40.7 ± 2.8^#^  t = 3.063  P = 0.018  g = 0.69  CI_95%_ = -0.32 – 1.70 | 40.4 ± 2.3^#^  P = 0.002  g = 1.29  CI_95%_ = 0.21 – 2.37 | 41.7 ± 3.0^#^  P = 0.001  g = 1.35  CI_95%_ = 0.26 – 2.43 | 39.0 ± 3.4 | 39.9 ± 3.4 |
|  |  |  |  |  |  |  |  |  |  |  |  |
| **t_D down_** ${\dot{\mathbf{V}}}_{\mathbf{O}_{\mathbf{2}}}$ **(s)** | 31.5 ± 1.7 | 29.9 ± 3.2 | 31.0 ± 1.7 | 30.8 ± 2.4 | 33.3 ± 1.6 |  | 38.6 ± 2.4^#^  t = 3.614  P = 0.009  g = 1.17  CI_95%_ = 0.11 – 2.23 | 46.1 ± 3.6^#^  P <0.001  g = 1.59  CI_95%_ = 0.47 – 2.71 | 41.1 ± 3.0^#^  P = 0.015  g = 1.37  CI_95%_ = 0.28 – 2.46 | 40.5 ± 3.2^#^  P = 0.007  g = 1.16  CI_95%_ = 0.10 – 2.22 | 38.1 ± 3.9 |
| **t_D down_** ${\dot{\mathbf{V}}}_{\mathbf{E}}$ **(s)** | 42.0 ± 2.7 | 41.1 ± 3.1 | 42.3 ± 3.2 | 41.8 ± 3.9 | 44.3 ± 3.6 |  | 45.7 ± 4.6 | 48.3 ± 4.5 | 49.4 ± 4.5 | 49.3 ± 5.1 | 44.6 ± 5.1 |
| **t_D down_** ${\dot{\mathbf{V}}}_{\mathbf{C}\mathbf{O}_{\mathbf{2}}}$ **(s)** | 42.1 ± 2.1 | 41.3 ± 3.0 | 43.4 ± 1.9 | 42.3 ± 3.4 | 43.1 ± 3.3 |  | 47.9 ± 3.0 | 53.4 ± 4.1^#^  P = 0.005  g = 1.14  CI_95%_ = 0.08 – 2.20 | 48.1 ± 3.1 | 48.6 ± 4.1 | 49.6 ± 3.6^##^  P = 0.034  g = 0.63  CI_95%_ = -0.37 – 1.63 |
| **t_D down_** $\boldsymbol{f}_{\mathbf{H}}$ **(s)** | 34.8 ± 2.8 | 29.8 ± 2.5 | 32.4 ± 2.4 | 35.6 ± 3.8 | 34.9 ± 2.7 |  | 45.7 ± 3.3^#^  t = 8.071  P <0.001  g = 1.18  CI_95%_ = 0.11 – 2.24 | 46.4 ± 3.5^#^  P <0.001  g = 1.84  CI_95%_ = 0.67 – 3.01 | 47.9 ± 3.8^#^  P <0.001  g = 1.62  CI_95%_ = 0.49 – 2.75 | 42.7 ± 3.8^#^  P = 0.046  g = 0.62  CI_95%_ = -0.38 – 1.62 | 44.6 ± 2.8^##^  P = 0.001  g = 1.18  CI_95%_ = 0.12 – 2.24 |

**Table 4:** Between protocols effect in blood lactate concentration ([La^-^]), rates of perceived exertion on a general (RPE_GEN_; Borg 6-20), respiratory and muscular (RPE_MUSC_ and RPE_RESP_; CR-10) values of each sinewave (sin) of CP-50_ex_ and CP_ex_.

Data are shown as mean ± standard error (SE). ^#^ p<0.05 vs CP-50_ex_.

| **Parameter** | **CP-50_ex_** | | | |  | **CP_ex_** | | | |
| --- | --- | --- | --- | --- | --- | --- | --- | --- | --- |
|  | **Sin1** | **Sin2** | **Sin3** | **SinLast** |  | **Sin1** | **Sin2** | **Sin3** | **SinLast** |
|  |  |  |  |  |  |  |  |  |  |
| **[La^-^] (mM)** | 3.0 ± 0.3 | 3.2 ± 0.4 | 2.9 ± 0.4 | 2.5 ± 0.4 |  | 4.2 ± 0.5^#^  P = 0.037  g = 1.03  CI_95%_ = -0.01 – 2.08 | 5.2 ± 0.6^#^  P = 0.011  g = 1.32  CI_95%_ = 0.24 – 2.40 | 5.2 ± 0.5^#^  P = 0.012  g = 1.61  CI_95%_ = 0.48 – 2.74 | 6.3 ± 0.5^#^  P <0.001  g = 2.57  CI_95%_ = 1.24 – 3.89 |
| **RPE_GEN 6-20_ (u.a.)** | 12.0 ± 0.6 | 12.9 ± 0.4 | 13.1 ± 0.4 | 18.3 ± 0.3 |  | 13.6 ± 0.5^#^  P = 0.048  g = 0.93  CI_95%_ = -0.10 – 1.97 | 15.0 ± 0.7^#^  P = 0.015  g = 1.27  CI_95%_ = 0.20 – 2.35 | 15.4 ± 0.6^#^  P = 0.008  g = 1.48  CI_95%_ = 0.38 – 2.59 | 18.4 ± 0.4 |
| **RPE_MUS 0-10_ (u.a.)** | 3.3 ± 0.2 | 3.9 ± 0.3 | 4.1 ± 0.3 | 8.3 ± 0.6 |  | 5.0 ± 0.4^#^  P = 0.010  g = 1.72  CI_95%_ = 0.57 – 2.86 | 5.9 ± 0.6^#^  P = 0.008  g = 1.38  CI_95%_ = 0.29 – 2.47 | 6.8 ± 0.6^#^  P <0.001  g = 1.88  CI_95%_ = 0.70 – 3.06 | 9.4 ± 0.4 |
| **RPE_RESP_ _0-10_ (u.a.)** | 2.6 ± 0.6 | 2.6 ± 0.5 | 3.0 ± 0.5 | 5.1 ± 0.4 |  | 3.2 ± 0.3 | 3.9 ± 0.5^#^  P = 0.039  g = 0.88  CI_95%_ = -0.15 – 1.90 | 4.8 ± 0.5^#^  P = 0.006  g = 1.18  CI_95%_ = 0.02 – 2.11 | 6.6 ± 0.5^#^ |

**Table 5:** Differences between the cycle-by-cycle and the overlapping analysis in midpoint (MP), amplitude (AMP) and time delay upward and downward MP crossing (t_D up_ and t_D down_, respectively) response of pulmonary oxygen uptake ($\dot{V}_{O_{2}}$), ventilation ($\dot{V}_{E}$), carbon dioxide production ($\dot{V}_{\mathrm{CO}_{2}}$) and heart rate ($f_{H}$) to each sinewave (sin) and to average sines of CP-50_ex_ (table 5a) and CP_ex_ (table 5b). Data are shown as mean ± standard error (SE). ^§^ p<0.05 *vs* average value

| **Table 5a** | **CP-50_ex_** | | | | |
| --- | --- | --- | --- | --- | --- |
|  | **Average** | **Sin1** | **Sin2** | **Sin3** | **SinLast** |
|  |  |  |  |  |  |
| **MP** ${\dot{\mathbf{V}}}_{\mathbf{O}_{\mathbf{2}}}$ **(ml∙min^-1^)** | 2826 ± 195 | 2776 ± 202^§^  t = 2.815  P = 0.026  g = 0.079  CI_95%_ = -0.01 – 0.162 | 2783 ± 201^§^  t = 2.116  P = 0.050  g = 0.070  CI_95%_ = -0.02 –0.16 | 2758 ± 189^§^  t = 2.285  P = 0.050  g = 0.116  CI_95%_ = -0.02 – 0.26 | 2900 ± 202^§^  t = 2.657  P = 0.033  g = 0.120  CI_95%_ = -0.01 – 0.25 |
| **MP** ${\dot{\mathbf{V}}}_{\mathbf{E}}$ **(l∙min^-1^)** | 78 ± 6 | 74 ± 7^§^  t = 2.385  P = 0.049  g = 0.194  CI_95%_ = -0.03 – 0.42 | 74 ± 6^§^  t = 2.883  P = 0.024  g = 0.182  CI_95%_ = -0.01 – 0.37 | 74 ± 6^§^  t = 2.640  P = 0.033  g = 0.191  CI_95%_ = -0.02 – 0.40 | 82 ± 7^§^  t = 4.028  P = 0.005  g = 0.193  CI_95%_ = 0.03 – 0.36 |
| **MP** ${\dot{\mathbf{V}}}_{\mathbf{C}\mathbf{O}_{\mathbf{2}}}$ **(ml∙min^-1^)** | 2661 ± 182 | 2681 ± 185 | 2652 ± 180 | 2611 ± 174^§^  t = 2.234  P = 0.06  g = 0.088  CI_95%_ = -0.02 – 0.20 | 2675 ± 187 |
| **MP** $\boldsymbol{f}_{\mathbf{H}}$ **(b∙min^-1^)** | 147 ± 5 | 138 ± 5^§^  t = 6.904  P <0.001  g = 0.569  CI_95%_ = 0.16 – 0.98 | 141 ± 5^§^  t = 3.753  P = 0.007  g = 0.366  CI_95%_ = 0.40 – 0.69 | 144 ± 5^§^  t = 2.331  P = 0.053  g = 0.237  CI_95%_ = -0.05 – 0.52 | 154 ± 5^§^  t = 4.795  P = 0.002  g = 0.502  CI_95%_ = 0.10 – 0.90 |
|  |  |  |  |  |  |
| **AMP** ${\dot{\mathbf{V}}}_{\mathbf{O}_{\mathbf{2}}}$ **(ml∙min^-1^)** | 488 ± 36 | 443 ± 28^§^  t = 2.481  P = 0.042  g = 0.432  CI_95%_ = -0.06 – 0.93 | 485 ± 35 | 461 ± 24 | 479 ± 51 |
| **AMP** ${\dot{\mathbf{V}}}_{\mathbf{E}}$ **(l∙min^-1^)** | 14.5 ± 1.3 | 11.6 ± 1.1^§^  t = 4.226  P = 0.004  g = 0.786  CI_95%_ = 0.12 – 1.45 | 13.5 ± 1.0 | 13.8 ± 1.3 | 15.3 ± 1.7 |
| **AMP** ${\dot{\mathbf{V}}}_{\mathbf{C}\mathbf{O}_{\mathbf{2}}}$ **(ml∙min^-1^)** | 487 ± 29 | 429 ± 27^§^  t = 3.915  P = 0.006  g = 0.689  CI_95%_ = 0.09 – 1.29 | 470 ± 27 | 457 ± 20^§^  t = 2.305  P = 0.050  g = 0.315  CI_95%_ = -0.06 – 0.69 | 493 ± 30 |
| **AMP** $\boldsymbol{f}_{\mathbf{H}}$ **(b∙min^-1^)** | 12.0 ± 1.1 | 12.0 ± 1.3 | 12.8 ± 1.3 | 12.0 ± 1.2 | 11.2 ± 1.2^§^  t = 2.362  P = 0.05  g = 0.218  CI_95%_ = -0.04 – 0.48 |
|  |  |  |  |  |  |
| **t_D up_** ${\dot{\mathbf{V}}}_{\mathbf{O}_{\mathbf{2}}}$ **(s)** | 31.0 ± 1.7 | 28.8 ± 3.1 | 30.1 ± 1.9 | 29.3 ± 2.3 | 32.8 ± 2.0 |
| **t_D up_** ${\dot{\mathbf{V}}}_{\mathbf{E}}$ **(s)** | 42.5 ± 2.5 | 41.8 ± 3.2 | 45.3 ± 3.5 | 43.8 ± 3.6 | 44.0 ± 3.1 |
| **t_D up_** ${\dot{\mathbf{V}}}_{\mathbf{C}\mathbf{O}_{\mathbf{2}}}$ **(s)** | 42.0 ± 1.9 | 41.6 ± 2.9 | 43.8 ± 1.9 | 41.9±3.2 | 42.1±3.4 |
| **t_D up_** $\boldsymbol{f}_{\mathbf{H}}$ **(s)** | 34.7 ± 2.9 | 30.6 ± 2.7 | 31.0 ± 2.2 | 36.5 ± 4.5 | 35.0 ± 3.1 |
|  |  |  |  |  |  |
| **t_D down_** ${\dot{\mathbf{V}}}_{\mathbf{O}_{\mathbf{2}}}$ **(s)** | 31.5 ± 1.7 | 29.9 ± 3.2 | 31.0 ± 1.7 | 30.8 ± 2.4 | 33.3 ± 1.6 |
| **t_D down_** ${\dot{\mathbf{V}}}_{\mathbf{E}}$ **(s)** | 42.0 ± 2.7 | 41.1 ± 3.1 | 42.3 ± 3.2 | 41.8 ± 3.9 | 44.3 ± 3.6 |
| **t_D down_** ${\dot{\mathbf{V}}}_{\mathbf{C}\mathbf{O}_{\mathbf{2}}}$ **(s)** | 42.1 ± 2.1 | 41.3 ± 3.0 | 43.4 ± 1.9 | 42.3 ± 3.4 | 43.1 ± 3.3 |
| **t_D down_** $\boldsymbol{f}_{\mathbf{H}}$ **(s)** | 34.8 ± 2.8 | 29.8 ± 2.5^§^  t = 2.564  P = 0.085  g = 0.63  CI_95%_ = -0.37 – 1.64 | 32.4 ± 2.4 | 35.6 ± 3.8 | 34.9 ± 2.7 |
|  |  |  |  |  |  |
| **Table 5b** | **CP_ex_** | | | | |
|  | **Average** | **Sin1** | **Sin2** | **Sin3** | **SinLast** |
|  |  |  |  |  |  |
| **MP** ${\dot{\mathbf{V}}}_{\mathbf{O}_{\mathbf{2}}}$ **(ml∙min^-1^)** | 3355 ± 185 | 3293 ± 180^§^  t = 2.990  P = 0.020  g = 0.111  CI_95%_ = -0.01 – 0.22 | 3359 ± 182 | 3354 ± 189 | 3410 ± 194^§^  t = 3.964  P = 0.005  g = 0.067  CI_95%_ = 0.01 – 0.13 |
| **MP** ${\dot{\mathbf{V}}}_{\mathbf{E}}$ **(l∙min^-1^)** | 105 ± 7 | 94 ± 7^§^  t = 9.231  P <0.001  g = 0.557  CI_95%_ = 0.18 – 0.94 | 100 ± 7^§^  t = 3.782  P = 0.007  g = 0.216  CI_95%_ = 0.024 – 0.408 | 105 ± 8 | 116 ± 7^§^  t = 6.836  P <0.001  g = 0.468  CI_95%_ = 0.13 – 0.81 |
| **MP** ${\dot{\mathbf{V}}}_{\mathbf{C}\mathbf{O}_{\mathbf{2}}}$ **(ml∙min^-1^)** | 3309 ± 182 | 3318 ± 177 | 3325 ± 174 | 3301 ± 179 | 3308 ± 193 |
| **MP** $\boldsymbol{f}_{\mathbf{H}}$ **(b∙min^-1^)** | 162 ± 4 | 156 ± 4^§^  t = 5.731  P = 0.001  g = 0.602  CI_95%_ = 0.15 – 1.06 | 160 ± 4^§^  t = 2.847  P = 0.025  g = 0.208  CI_95%_ = -0.01 – 0.425 | 163 ± 4 | 167 ± 4^§^  t = 6.594  P <0.001  g = 0.474  95% CI = 0.13 – 0.82 |
|  |  |  |  |  |  |
| **AMP** ${\dot{\mathbf{V}}}_{\mathbf{O}_{\mathbf{2}}}$ **(ml∙min^-1^)** | 304 ± 11 | 311 ± 16 | 311 ± 12 | 312 ± 17 | 287 ± 15 |
| **AMP** ${\dot{\mathbf{V}}}_{\mathbf{E}}$ **(l∙min^-1^)** | 14.0 ± 0.8 | 11.7 ± 1.3 | 12.4 ± 1.0 | 12.9 ± 1.0 | 16.4 ± 1.5^§^  t = 2.308  P = 0.050  g = 0.534  CI_95%_ = 0.11 – 1.18 |
| **AMP** ${\dot{\mathbf{V}}}_{\mathbf{C}\mathbf{O}_{\mathbf{2}}}$ **(ml∙min^-1^)** | 389 ± 13 | 398 ± 18 | 388 ± 12 | 388 ± 16 | 376 ± 21 |
| **AMP** $\boldsymbol{f}_{\mathbf{H}}$ **(b∙min^-1^)** | 8.2 ±1.1 | 8.5 ±1.1 | 8.5 ± 1.1 | 8.4 ± 1.2 | 7.7 ± 1.1 |
|  |  |  |  |  |  |
| **t_D up_** ${\dot{\mathbf{V}}}_{\mathbf{O}_{\mathbf{2}}}$ **(s)** | 35.1 ± 1.8 | 39.6 ± 2.7 | 33.5 ± 3.7 | 37.0 ± 2.3 | 34.2 ± 1.8 |
| **t_D up_** ${\dot{\mathbf{V}}}_{\mathbf{E}}$ **(s)** | 48.8 ± 4.4 | 54.2 ± 4.8^§^  t = 2.137  P = 0.07  g = 0.09  CI_95%_ = -0.89 – 1.07 | 48.6 ± 4.0 | 47.1 ± 4.3 | 44.0 ± 4.6^§^  t = 2.483  P = 0.042  g = 0.18  CI_95%_ = -0.80 – 1.16 |
| **t_D up_** ${\dot{\mathbf{V}}}_{\mathbf{C}\mathbf{O}_{\mathbf{2}}}$ **(s)** | 48.2 ± 2.9 | 50.9 ± 3.1 | 47.4 ± 3.7 | 47.8 ± 3.5 | 48.4 ± 3.3 |
| **t_D up_** $\boldsymbol{f}_{\mathbf{H}}$ **(s)** | 40.7 ± 2.8 | 40.4 ± 2.3 | 41.7 ± 3.0 | 39.0 ± 3.4 | 39.9 ± 3.4 |
|  |  |  |  |  |  |
| **t_D down_** ${\dot{\mathbf{V}}}_{\mathbf{O}_{\mathbf{2}}}$ **(s)** | 38.6 ± 2.4 | 46.1 ± 3.6^§^  t = 3.906  P = 0.006  g = 0.81  CI_95%_ = -0.21 – 1.83 | 41.1 ± 3.0 | 40.5 ± 3.2 | 38.1 ± 3.9 |
| **t_D down_** ${\dot{\mathbf{V}}}_{\mathbf{E}}$ **(s)** | 45.7 ± 4.6 | 48.3 ± 4.5 | 49.4 ± 4.5 | 49.3 ± 5.1 | 44.6 ± 5.1 |
| **t_D down_** ${\dot{\mathbf{V}}}_{\mathbf{C}\mathbf{O}_{\mathbf{2}}}$ **(s)** | 47.9 ± 3.0 | 53.4 ± 4.1^§^  t = 3.002  P = 0.02  g = 0.51  CI_95%_ = -0.48 – 1.51 | 48.1 ± 3.1 | 48.6 ± 4.1 | 49.6 ± 3.6 |
| **t_D down_** $\boldsymbol{f}_{\mathbf{H}}$ **(s)** | 45.7 ± 3.3 | 46.4 ± 3.5 | 47.9 ± 3.8^§^  t = 2.003  P = 0.08  g = 0.21  CI_95%_ = -0.77 – 1.91 | 42.7 ± 3.8 | 44.6 ± 2.8 |
